# Metagenomic features of bioburden serve as outcome indicators in combat extremity wounds

**DOI:** 10.1038/s41598-022-16170-x

**Published:** 2022-08-15

**Authors:** Aram Avila-Herrera, James B. Thissen, Nisha Mulakken, Seth A. Schobel, Michael D. Morrison, Xiner Zhou, Scott F. Grey, Felipe A. Lisboa, Desiree Unselt, Shalini Mabery, Meenu M. Upadhyay, Crystal J. Jaing, Eric A. Elster, Nicholas A. Be

**Affiliations:** 1 Computing and Global Security Directorates, Lawrence Livermore National Laboratory, Livermore, CA USA; 2 Physical and Life Sciences Directorate, Lawrence Livermore National Laboratory, Livermore, CA USA; 3 Surgical Critical Care Initiative (SC2i), Uniformed Services University of the Health Sciences (USUHS), Bethesda, MD USA; 4 The Henry M. Jackson Foundation for the Advancement of Military Medicine, Inc., Bethesda, MD USA; 5 Present Address: Department of Statistics, University of California, Davis, CA USA; 6 Present Address: Translational Genomics, Q2 Solutions, Durham, NC USA; 7 Walter Reed National Military Medical Center, Bethesda, MD USA

**Keywords:** Microbiology, Clinical microbiology, Microbial communities, Infectious-disease diagnostics, Infectious diseases, Trauma, Genome informatics, Bioinformatics, Microbiology techniques, Sequencing, Computational biology and bioinformatics

## Abstract

Battlefield injury management requires specialized care, and wound infection is a frequent complication. Challenges related to characterizing relevant pathogens further complicates treatment. Applying metagenomics to wounds offers a comprehensive path toward assessing microbial genomic fingerprints and could indicate prognostic variables for future decision support tools. Wound specimens from combat-injured U.S. service members, obtained during surgical debridements before delayed wound closure, were subjected to whole metagenome analysis and targeted enrichment of antimicrobial resistance genes. Results did not indicate a singular, common microbial metagenomic profile for wound failure, instead reflecting a complex microenvironment with varying bioburden diversity across outcomes. Genus-level *Pseudomonas* detection was associated with wound failure at all surgeries. A logistic regression model was fit to the presence and absence of antimicrobial resistance classes to assess associations with nosocomial pathogens. *A. baumannii* detection was associated with detection of genomic signatures for resistance to trimethoprim, aminoglycosides, bacitracin, and polymyxin. Machine learning classifiers were applied to identify wound and microbial variables associated with outcome. Feature importance rankings averaged across models indicated the variables with the largest effects on predicting wound outcome, including an increase in *P. putida* sequence reads. These results describe the microbial genomic determinants in combat wound bioburden and demonstrate metagenomic investigation as a comprehensive tool for providing information toward aiding treatment of combat-related injuries.

## Introduction

Combat trauma patients from U.S. military conflicts of the past several decades have shown increasingly severe injuries. In 2005–2009, the Joint Theater Trauma Registry recorded 17,177 wounds associated with musculoskeletal injuries in service members deployed to Iraq and Afghanistan^1^. These injuries are often devastating, and their characteristics are fundamentally distinct from those observed in civilians^2^. The nature of trauma from blast injuries and high-energy ballistics is extensive and pushes the limit of patient physiology, survivability, and treatment technology.

Injury mechanisms and the extent of trauma facilitate wound microbial contamination. Up to 52% of patients with amputations from combat trauma may be diagnosed with some form of extremity wound infections; this may increase to approximately 68% in above knee amputations^3^. Large wound surface area and the exposure to environmental contaminants may increase the likelihood of microbial complications.

In extremity wound treatment, bioburden microbial identification is typically performed using standard bacteriology culture; however, culture-based analyses underestimate microbial diversity and burden^4^. Studies in chronic wounds show that a comprehensive understanding of bioburden is critical to interpreting healing responses^5,6^. Culture-independent methods identify microbial species not indicated via standard bacteriology and suggest that distinct microbial signatures may associate with specific outcomes^7^, particularly with nosocomial pathogens^8^. A frequent complication with such pathogens is the incidence of multidrug resistant organisms (MDRO), which remained a concern in the treatment of wounded warriors during Operation Iraqi Freedom (OIF) and Operation Enduring Freedom (OEF)^9^. MDRO infection represents a substantial challenge for antibiotic treatment, and in some cases, despite best efforts, leads to outgrowth of resistant subpopulations. Additionally, emerging unknown resistant bacteria may be undetected by traditional culture, as only a fraction of viable microorganisms in a sample may be accurately cultivated for testing. Improved metrics of antibiotic resistance in wound infection could guide treatment in these cases.

Metagenomic sequencing can provide a comprehensive analysis of microbial composition. Some such efforts employ a targeted amplicon-based approach via amplification of bacterial 16S rRNA gene regions, followed by sequencing. This method is well-validated, but limits resolution, does not capture data from non-bacterial (fungal, viral, protozoan) entities^10^, and only captures taxonomic information, as opposed to gene level detail. By contrast, an approach employing shotgun whole metagenome sequencing is untargeted^7,11–13^. Whole metagenome data is also more sensitive, revealing lower abundant microbes that can be critical to distinguishing experimental states^14^, and facilitates higher taxonomic resolution with a larger number of detected taxa^15^. Assessing individual microbial genes, however, requires more detailed (i.e., deeper) sequencing of samples. Targeted sequencing can be employed for enrichment of defined genomic regions. One such method performs highly multiplexed pre-amplification prior to sequencing and could detect microbial genomic resistance determinants.

These approaches hold promise for informing care in situations where the relevant pathogen is difficult to culture or the gene of interest is present in low abundance or low copy number, for instance detection of antimicrobial resistance loci in low biomass samples^16^ or detection of low-abundant anaerobic species^17^. A further challenge is the interpretation of microbial metagenomic data and assessment of its predictive value. Previous studies have demonstrated the promise of employing machine learning and other biostatistical techniques for predicting the outcome of trauma from biomolecular features^18–20^. Machine learning methods have been used to process multi-omic datasets for identification of features predictive of trauma^21^ and have associated viral micro-RNA detection with injury outcome^22^.

We applied whole metagenome sequencing for microbial identification and a targeted amplification panel for the identification of antimicrobial resistance (AMR)-associated genes in combat-related extremity wounds. We assessed taxonomic composition of whole metagenome data and genomic AMR signature profiles in the context of clinical observations, treatment, and patient outcomes, revealing metagenomic features that could guide future clinical practice.

## Results

### Taxonomic characteristics of microbial bioburden in combat wounds

**Figure 1:**
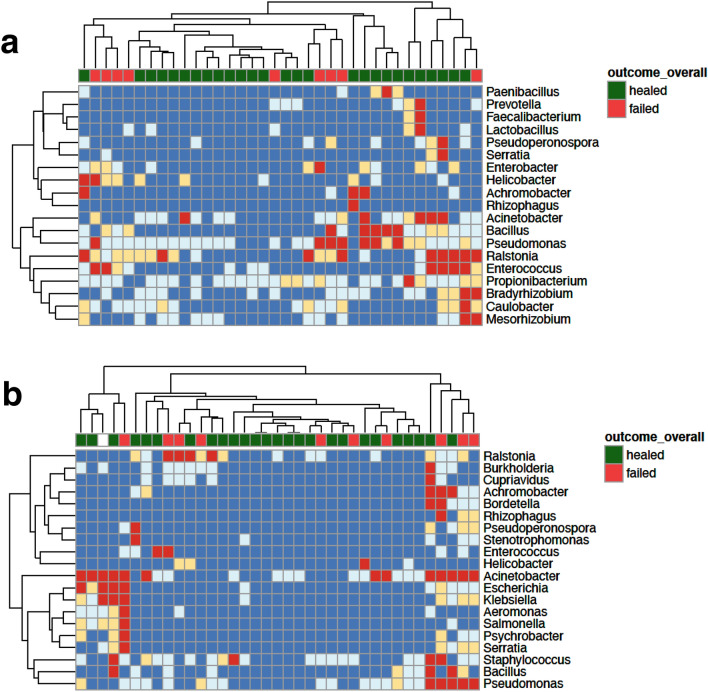
Microbial profile as determined by whole metagenome sequencing and read-based taxonomic analysis. Shotgun metagenomic sequencing and taxonomic classification of sequence was performed to assess the microbial wound bioburden for (**a**) initial and (**b**) final (day of delayed wound closure) wound effluent specimens. Read abundance relative to total sequence content, including human-derived background sequence data, is binned and shown as follows: Only microbial genera for which at least one analyzed specimen demonstrated a relative abundance for that genus of > 1E−5 are shown. Abundance values were binned on a log scale. dark blue: < 1E−7, light blue: ≥ 1E−7 & < 1E−6, yellow: ≥ 1E−6 & < 1E−5, red: ≥ 1E−5. Wound outcome is annotated along the top of the heatmap, with green indicating successful healing; red indicating failed; white indicating outcome not available.

Bioburden was assessed via whole metagenome sequencing and read-based taxonomic assignment, examining DNA sequence relative abundance for wound tissue and effluent specimens collected at initial, intermediate, and final time points. Distinct clusters of failed wounds were observed, as defined by profiling higher abundant microbial species in effluent (Fig. 1). At the initial time point, larger clusters of samples were observed, while the final time point yielded smaller, more distributed clusters of samples. The absence of a single cluster of failed wounds indicates that one microbial profile does not associate exclusively with wound failure, either at initial (Fig. 1a) or final (Fig. 1b) specimen collections. A complete taxonomic profile is provided in Supplementary Figure S1, with associated sequence read counts provided as a tabular Supplementary File.

Dimensionality reduction based on microbial profile was performed to visualize whether wound specimens clustered in salient patterns or according to labels such as outcome or specimen type (Supplementary Figure S2). Specimens derived from tissue samples of wounds that failed to heal appear to have smaller within-group variation (i.e., lower beta-diversity) than their successfully healed counterparts, suggesting a common signature that may be exploited by machine learning methods. However, this is not apparent in samples derived from effluent. In fact, in effluent samples, the between sample distances increase at intermediate time points compared to the initial time point and remain so at the final. It is likely that the multifactorial nature of wound healing and sample collection will be challenging to represent in two dimensions and requires more complex analyses and modeling. These observations further motivated subsequent variable selection and modeling assessments in the current study.

Co-occurrence of microbial genus detection was examined in wound effluent (Supplementary Table S1). Several genus pairs correlated significantly (Fisher’s exact test *P* < 0.01) when examining intermediate wound effluent specimens, including *Escherichia* with both *Acinetobacter* and *Serratia*, *Streptococcus* with both *Enterococcus* and *Staphylococcus*, *Staphylococcus* with *Enterococcus*, and *Achromobacter* with *Bacillus.* A total of 45 out of 59 wounds examined in this correlation analysis had specimens collected at intermediate time points produced during the series of debridement surgeries. These were obtained between the initial debridement surgery and the day of wound closure. Of those 45, the majority had one intermediate specimen. For the remainder, sample quantity differed due to varying treatment plans, and thus differing time-to-closure between patients (samples available for wounds with intermediate time points: min = 1, max = 10, median = 1, mean = 2.2). When examining final pre-closure specimens only (N = 36 wounds), the significant associations observed included *Achromobacter* with *Bordetella* and *Acinetobacter* with *Pseudomonas*. Odds ratio estimates and significance levels for all detected genera and statistical treatments are given in Supplementary Figure S3. These results indicate that certain microbial genera co-occur in combat wounds, but that wound microbial profile does not exclusively segregate according to wound outcome.

### Comparative analysis of metagenomic and quantitative bacteriology results

Quantitative bacteriology has for several decades been considered a relevant tool within the armament of clinical microbiology laboratories, particularly for assessing bacterial burden toward informing wound care^23^. A comparative analysis was performed to determine the degree of consistency and data concordance between metagenomic and culture-based bacteriology. The positive, negative, and overall percent agreement (PA) were calculated using 66 tissue specimens with data available from both methods. At genus-level resolution, quantitative bacteriology identified nine genera across 65/66 samples (98.5%) (Table 1). Metagenomic sequencing matched quantitative bacteriology at 83.1% and identified a genus in one specimen where quantitative bacteriology did not. The overall agreement rate of metagenomic sequencing with quantitative bacteriology was 81.8%. The most prevalent genus identified via bacteriology was *Acinetobacter* (57.6% of samples). Metagenomic analysis identified this genus in 100% of bacteriology-positive samples. *Enterococcus* occurred in 21.2% of samples; metagenomic analysis positively identified this genus in 50% of these cases. *Achromobacter* was identified in 7.6% of the samples; metagenomic analysis positively identified this genus in 40% of these cases. Six additional genera were identified by quantitative bacteriology in two or fewer samples. Metagenomic sequencing exhibited a 100% positive PA in these cases.

**Table 1:** Concordance between microbial genus-level detection by quantitative bacteriology and metagenomic sequencing.

Genus	QuantBAC Prevalence	Metagenomic sequencing percent positive agreement
Overall	65/66 (98.5%)	54/65 (83.1%) [72.2–90.3%]
Acinetobacter	38/66 (57.6%)	38/38 (100%) [90.8–100%]
Enterococcus	14/66 (21.2%)	7/14 (50%) [26.8–73.2%]
Achromobacter	5/66 (7.6%)	2/5 (40%) [11.8–76.9%]
Pseudomonas	2/66 (3%)	2/2 (100%) [34.2–100%]
Staphylococcus	2/66 (3%)	2/2 (100%) [34.2–100%]
Bacillus	1/66 (1.5%)	1/1 (100%) [20.7–100%]
Citrobacter	1/66 (1.5%)	1/1 (100%) [20.7–100%]
Enterobacter	1/66 (1.5%)	1/1 (100%) [20.7–100%]
Escherichia	1/66 (1.5%)	1/1 (100%) [20.7–100%]

Concordance was assessed according to prevalence, with positive percent agreement estimates used to compare genus detection from metagenomic sequencing to quantitative bacteriology, applied here as a non-reference standard. Note: except for one case, these tests did not have a Negative result to tabulate.

Similar results were observed at species-level resolution, where quantitative bacteriology identified eight species across 57/66 samples (86.4%). Metagenomic sequencing positively matched quantitative bacteriology in 78.9% of samples, identifying at least one species in 66.7% (6/9) samples where quantitative bacteriology yielded negative species-level results. The overall species agreement rate was 77.3% (Supplementary Table S2). Overall, comparison of bacteriology and metagenomic sequencing show high positive PA (72–90% for genus and 67–88% for species).

### Bioburden taxonomic diversity in wounds

**Figure 2:**
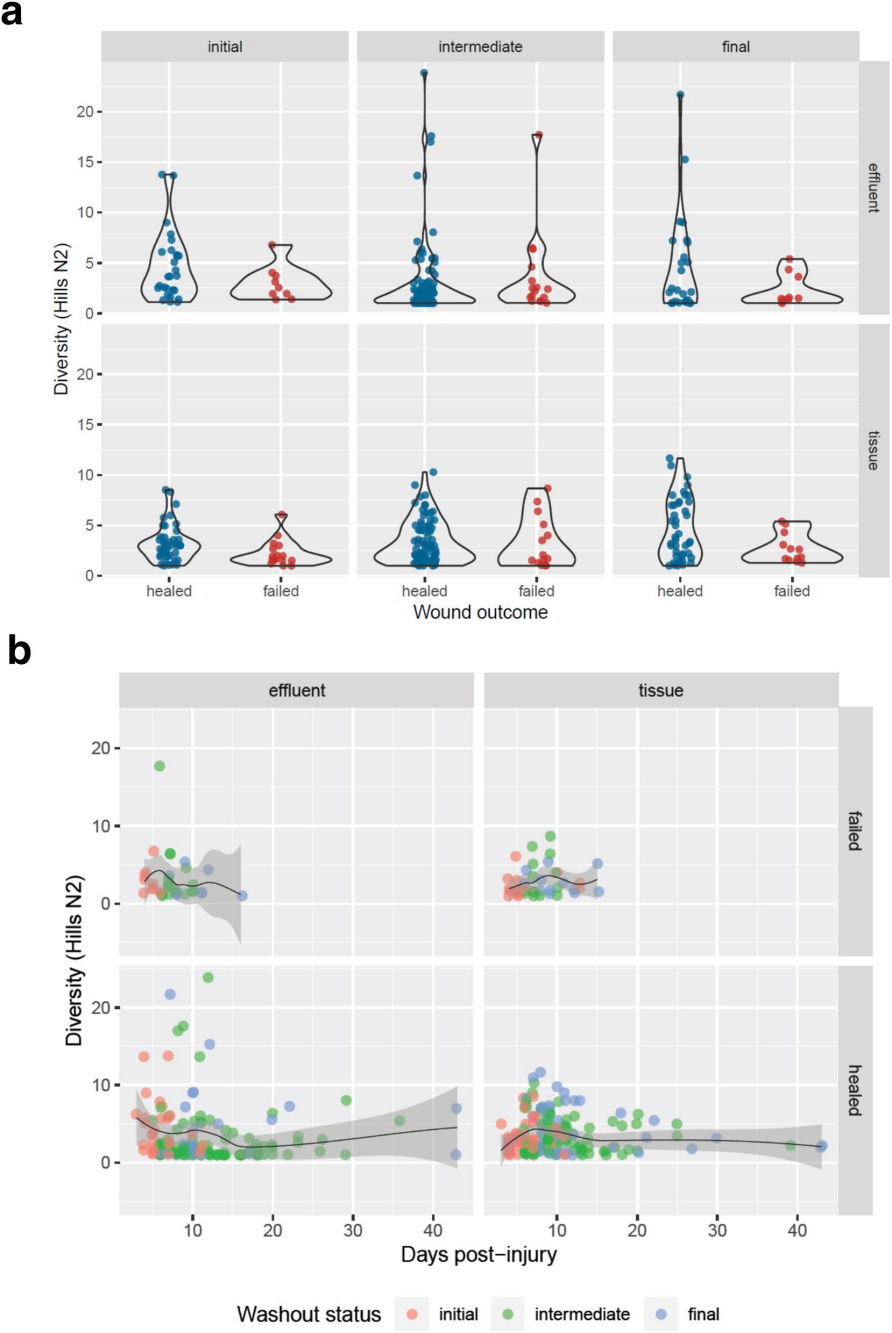
Alpha diversity of bioburden according to wound status. Metagenomic sequence data from wound specimens was used to calculate bioburden diversity through assessment of richness. (**a**) Microbial taxonomic diversity as represented by Hill’s N_2_ (reciprocal Simpson index) at the genus level, per sample, in distinct samplings from wounds. Diversity trends are shown in comparison to wound outcome. (**b**) Effective number of genera (Hill’s N_2_) shown according to increasing number of days post injury. Specimen timepoint category is indicated via color of the corresponding point. Shaded area surrounding loess trendlines indicates 95% confidence interval about the average N_2_ genera.

Microbial diversity metrics were examined in the context of wound outcome (healing success vs. failure) and number of days post-injury for tissue and effluent specimens (Fig. 2). Alpha diversity was quantified at the genus level using the Hill numbers N_0_, N_1_, and N_2_ (effective number of genera). Alpha diversity was lower in specimens from failed wounds at final (pre-closure) timepoints, relative to those from healed wounds, in both tissue (healed [43 samples]: N_2_ = 4.6 ± 0.5; failed [12 samples]: N_2_ = 2.7 ± 0.4 [mean ± SEM]) and effluent (healed [26 samples]: N_2_ = 4.6 ± 1.0; failed [10 samples]: N_2_ = 2.3 ± 0.5 [mean ± SEM]) specimens. This distinction was not, however, statistically significant (tissue: *P* = 0.08; effluent: *P* = 0.40 [Wilcoxon rank sum test]), as low sample quantities were available from this unique cohort (Fig. 2a). No differences in richness were observed across sample categories (Supplementary Figure S4).

Hill’s N_2_ was examined using the number of days post-injury for each specimen time point (Fig. 2b). Samples with elevated diversity were observed more frequently at timepoints proximal to injury. Due to the severe nature of combat polytrauma, injured patients receive a wide range of potential interventions and treatments. Although impacts of each of these interventions on microbial diversity are of interest, they were not examined in the current study due to limited sample availability across intervention groups, restricting the capacity to make statistically robust observations. A summary of the distribution of genus level richness across wound and sample types is given in Supplementary Table S3. The impact of further interventions will be the subject of future study.

### Prevalence of bioburden constituents across distinct wound outcomes

**Figure 3:**
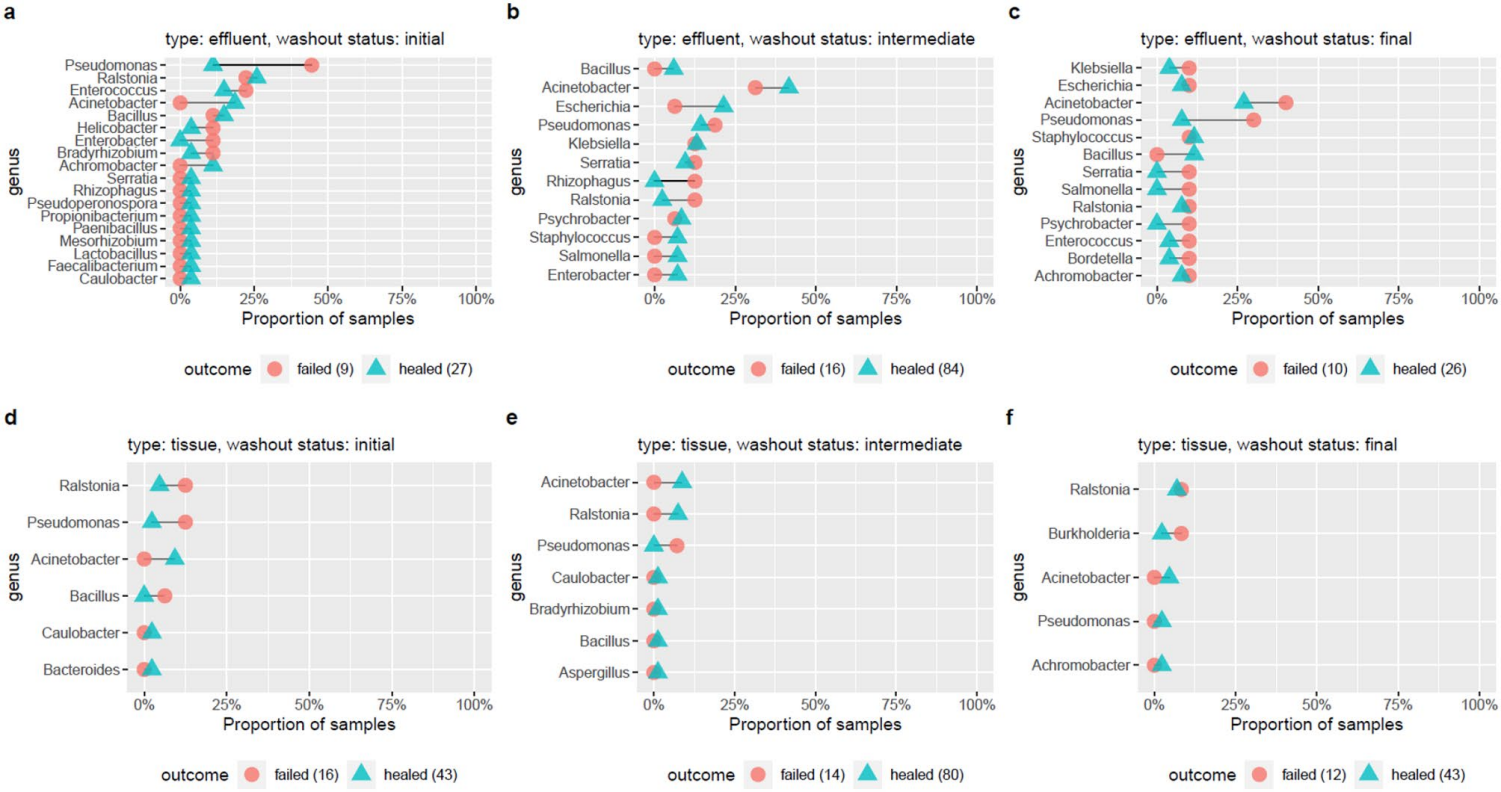
Prevalence of microbial genera in samples derived from healed or failed wounds. Prevalence of detection of microbial genera at defined thresholds was calculated for wounds that either healed successfully or failed to heal. Prevalence is shown as a proportion of total healed or failed samples for each given sample category, where effluent samples are shown in panels (**a-c**), and tissue samples are shown in panels (**d–f)**. Samples are shown for initial (**a**,**d**), intermediate (**b**,**e**), and final (**c**,**f**) collection timepoints. Individually significant (*P* < 0.05) distinctions are shown with a bolded edge. Depending on time-to-closure for each wound, a variable number of samples were available for intermediate samplings. A total of 100 effluent samples (across 46 wounds) and 94 tissue samples (across 49 wounds) were examined.

Prevalence of microbial taxa (presence defined as relative abundance > 1 × 10^−5^, top 20 genera selected) was examined across initial, intermediate, and final specimen time points (Fig. 3). Each wound is represented by a single initial and single final assessment. In this analysis of intermediate specimens, a total of 100 effluent samples (across 46 wounds) and 94 tissue samples (across 49 wounds) were employed.

At all time points, *Pseudomonas* were detected in wound effluent at a higher prevalence in samples from failed wounds relative to healed wounds (4/9 *Pseudomonas* positive from failed wounds versus 3/27 *Pseudomonas* positive from healed wounds, odds ratio [OR] = 5.98 [0.76–55.28], *P* = 0.05) (Fig. 3a–c). Prevalence of *Rhizophagus* was significantly higher in intermediate samples from failed wounds compared to healed wounds (2/16 failed, 0/84 healed, *P* = 0.02). *Acinetobacter* prevalence was higher in effluent from failed wounds, relative to healed wounds, at the final timepoint (detection in 4/10 failed specimens and 7/26 healed specimens, OR = 1.78 [0.28–10.53]). Sample numbers were not, however, sufficient to assign statistical significance (*P* = 0.45). No statistically significant distinctions in prevalence were observed in tissue samples (Fig. 3d–f). The observation of differential prevalence of microbial taxa between distinct wound outcomes, as revealed by metagenomic techniques, suggests that metagenomic features could be predictive of clinical outcomes, dependent on the taxa of interest.

### AMR genomic signature detection and nosocomial pathogen detection

A targeted amplification panel was applied to evaluate AMR genomic signatures. To calibrate the sequence read levels assigned to “presence” or “absence” of a given AMR gene, this panel was applied to negative (human reference gDNA only) and positive (*Acinetobacter baumannii* and *Pseudomonas aeruginosa*) controls. Thresholds were chosen to achieve zero gene detection events in two human reference gDNA-only samples. (Supplementary Figure S5). Specificity was assessed by comparing detected genes to ground truth in reference sequences for positive controls. All anticipated genes in the *P. aeruginosa* reference strain and 5/7 in the *A. baumannii* reference strain were detected (Supplementary Figure S6). It is possible that the two undetected *A. baumannii* genes were not identified due to similarity with genes from the same family, resulting in detection of another gene in that family. The beta-lactamase gene *blaADC-15* was anticipated to be present based on the *A. baumannii* reference sequence but was not detected by the targeted sequencing method. Several other beta-lactamases similar to *blaADC-15* were, however, detected, including *blaADC-10* (89% identical) and *blaADC-25* (90% identical). It is possible that amplicons corresponding to blaADC-15 were either not assigned to this reference gene due to SNPs and insertions in the strain employed for experimental testing, or were assigned to the noted, similar beta-lactamase genes.

In samples from both effluent and tissue, many wounds had no detectable AMR genes following threshold application. The first four timepoints (A–D) spanned the first 18 days of treatment. In this timeframe, we detected genomic AMR signatures for an average of two resistance classes per effluent sample. To determine whether detection of resistance to specific antibiotic classes associated significantly with detection of specific wound-relevant nosocomial pathogens, models were constructed to predict the presence or absence of signatures for resistance to each antimicrobial class, using abundance of nosocomial pathogen sequence as predictive features. Eighteen logistic regressions were independently fit to the presence or absence of AMR gene signatures, one for each antimicrobial resistance class. Each class was indicated as present if at least one associated AMR gene signature from that class was present. The analysis accounted for sample type (tissue versus effluent) and employed, as a proxy for nosocomial pathogen abundance as predictors, the log_2_ read counts for taxonomically assigned sequence reads. Each model predicts the detection of resistance to one of the given antimicrobial categories based on the number of reads mapped to each nosocomial pathogen. The model coefficients represent the increase in log odds of resistance per each log_2_ increase in reads (i.e., twofold increase or doubling). Models for 12/18 AMR class models fit sufficiently to allow coefficient interpretation according to a Likelihood Ratio Test of null deviance versus fitted deviance (Supplementary Table S4). Each association was examined for both polarity and effect size (Supplementary Figure S7).

**Figure 4:**
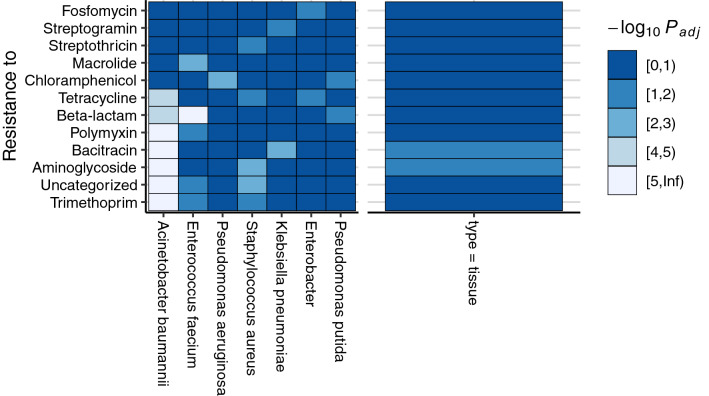
Association of AMR signature classes with detection of nosocomial pathogens. Logistic regression models were built to determine whether detection of defined genomic resistance signatures associates with metagenomic read abundance of specific genera corresponding to nosocomial pathogens. Resistance to drug classes are rows and nosocomial pathogens are columns. The sample collection type is included as an independent variable in each model with the type = tissue as compared to type = effluent shown as a separate column for reference. Significance (false discovery rate adjusted P-value) is indicated through color fill on a − log10 scale.

Out of the 12 interpretable models for AMR classes, 9 had at least one large positive effect from a nosocomial pathogen, such that the odds ratio estimate of detecting resistance genes of the given class is greater than 1.5 (50% increase) for each doubling of pathogen read counts. Out of the 7 nosocomial pathogens, 5 had a large positive effect on at least one AMR class. Out of the 84 associations tested, 14 had large positive effects (Table 2). *A. baumannii* metagenomic sequence abundance associated with the most AMR classes (associated in 5 models). It associated significantly with trimethoprim, aminoglycoside, bacitracin, polymyxin, and uncategorized resistance classes (all P_FDR_ <  < 0.001) (Fig. 4). *Enterobacter* positively associated with Fosfomycin resistance (P_FDR_ = 0.014). *Klebsiella pneumoniae* positively associated with bacitracin resistance (P_FDR_ = 0.009)*.* Two models (for Bacitracin and Aminoglycoside resistance) had significant effects from specimen type, potentially due to the difference in DNA content affecting the ability to detect resistance genes.

**Table 2:** Association of AMR signature classes with nosocomial pathogen detection in wound samples.

Resistance category	Nosocomial pathogen	Odds ratio [± SE]
*Bacitracin*	*Klebsiella pneumoniae*	3.45 [2.35, 5.06]
*Streptogramin*	*Klebsiella pneumoniae*	2.80 [1.87, 4.19]
*Fosfomycin*	*Enterobacter*	2.25 [1.62, 3.11]
*Chloramphenicol*	*Pseudomonas aeruginosa*	1.95 [1.65, 2.32]
*Bacitracin*	*Acinetobacter baumannii*	1.94 [1.76, 2.14]
*Aminoglycoside*	*Staphylococcus aureus*	1.85 [1.53, 2.23]
*Aminoglycoside*	*Acinetobacter baumannii*	1.75 [1.62, 1.89]
*Unassigned genes*	*Staphylococcus aureus*	1.68 [1.45, 1.94]
*Trimethoprim*	*Acinetobacter baumannii*	1.64 [1.54, 1.75]
*Unassigned genes*	*Acinetobacter baumannii*	1.63 [1.53, 1.73]
*Trimethoprim*	*Staphylococcus aureus*	1.61 [1.38, 1.87]
*Polymyxin*	*Acinetobacter baumannii*	1.60 [1.49, 1.71]
*Chloramphenicol*	*Klebsiella pneumoniae*	1.60 [1.28, 2.00]
*Streptothricin*	*Staphylococcus aureus*	1.50 [1.28, 1.76]

Logistic regression models were constructed for classes of antimicrobial resistance genes detected via targeted sequencing in wound samples. The observed effects for resistance are shown, odds ratio > 1.5.

### Association of AMR genomic signature detection with treatment regimen and patient status

**Figure 5:**
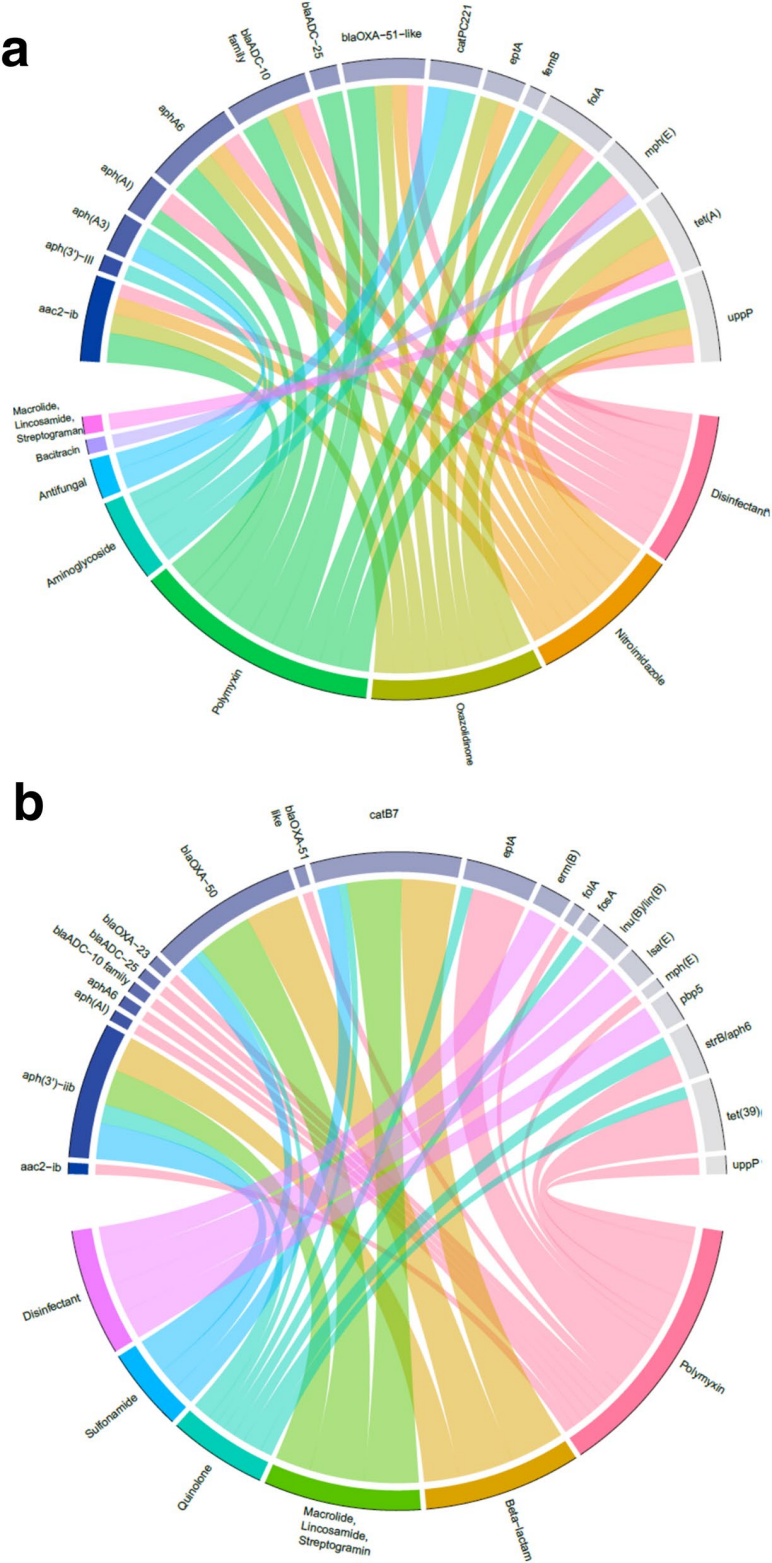
Association between antimicrobial treatment regimen and detection of antimicrobial resistance gene signatures in wound effluent samples. Mutual information analysis was applied to compare detection of genomic signatures for antimicrobial resistance with drug classes administered. Detection of resistance gene signatures is shown in samples obtained from both (**a**) healed and (**b**) failed wounds, to examine distinctions in associations across these patient subgroups.

AMR gene/class detection was also examined in the context of antimicrobial treatment regimen. Mutual information (MI) was used to quantify the association between AMR signature detection and categorical treatment variables. Analysis was restricted to effluent samples due to few AMR genes observed in tissue samples. MI was calculated for effluent samples derived from either healed or failed wounds to determine if associations differed between outcomes. In both healed and failed wounds, significant associations were observed between AMR genes and administration of polymyxins, macrolide/lincosamide/streptogramin (MLS), and disinfectants (Fig. 5). In healed wounds only, significant associations were observed between AMR genes and administration of aminoglycosides, antifungals, bacitracin, oxazolidinones, and nitroimidazoles. Aminoglycoside resistance genes detected included aminoglycoside phosphotransferase *aphA-3*. In failed wounds only, significant associations were observed between AMR genes and administration of beta-lactams, quinolones, and sulfonamides. Several of the strongest associations in failed wounds were observed with administration of beta-lactams, including the beta-lactamase *blaOXA-50*.

It is expected that antimicrobial exposure will exert selective pressure with respect to the prevalence of antimicrobial resistance genes within a microbial population. This study examined this dynamic in the context of a unique set of injuries and a microbial population restricted to the local environment of the wound, extending the association of such genes with the outcome of the injury as a whole. These results allow that injury outcomes could depend on interactions between AMR gene classes and treatment. To evaluate, prevalence of AMR class detection was examined in healed vs. failed wounds (Supplementary Figure S8A). Few significant differences were observed in the prevalence of resistance class signatures between healed and failed wounds, apart from intermediate effluent samples, where tetracycline and polymyxin resistance classes were significantly more prevalent in healed wounds. In final effluent samples, aminoglycoside and beta-lactam resistance classes were observed more frequently in failed wounds (difference in prevalence > 25%); however, this difference was not statistically significant. Thus, while distinctions exist between healed and failed wounds in the associations between antimicrobial administration and AMR gene detection, the prevalence of various AMR gene classes did not differ significantly between wound outcomes.

Given the potential of microbes to exert systemic effects, AMR class prevalence was compared with incidence of multiple organ dysfunction syndrome (MODS) (Supplementary Figure S8B), in which homeostasis is no longer maintained following acute injury. The prevalence of resistance was significantly higher in samples from patients with MODS, including aminoglycoside resistance in effluent at initial time points and four resistance categories in effluent at intermediate time points. Analysis was also performed according to injury type (amputation, open fracture, soft tissue injury [STI]) (Supplementary Figure S8C). Significant differences in AMR class prevalence were observed in specimens collected at intermediate and final time points, where in each significant case, AMR class prevalence (tetracyclines, polymyxins) was higher in STIs. In the case of tetracycline, prevalence of resistance genes for this class was ≥ 50% higher in STIs relative to open fractures.

### Construction of predictive wound healing models using metagenomic features

Our observations point toward possible predictive utility of metagenomic variables. Initial efforts at clustering (Fig. 1) did not reveal a clear, unambiguous microbial profile associated with wound healing failure. To examine relevant metagenomic features in more detail and assess clinical utility, machine learning classification was performed to identify whether microbial variables are predictive in combination with clinical variables and compare the relative performance of models for portending outcome. Feature categories included clinical covariates (*e.g.,* APACHE II score, wound size) and the following microbial features: targeted sequencing-derived AMR presence-absence (*e.g.,* tetracycline resistance gene present/absent) and shotgun metagenomic sequencing derived features (log read counts assigned to nosocomial pathogens, genus diversity metrics). Full- and *sans*-microbial feature sets were evaluated in separate training analyses.

Samples from this study were obtained from separate timepoints, as patients underwent a series of debridement surgeries before delayed wound closure. The outcome variable of interest (i.e., healing success vs. failure), however, is only measured after the final time point. Due to a focus on wound outcome, data from the final pre-closure specimen (tissue and effluent) were employed. Hierarchical clustering showed that samples with failed outcomes had a nearest neighbor with a failed outcome less than half the time. Additionally, many features were absent between samples and clustered together by observation type (e.g., physical wound attributes, taxonomy from whole genome sequencing, and antibiotic-related variables) (Supplementary Figure S9), implying that either (1) most measured variables are combining via physiologically complex processes opaque to this analysis or (2) variables will have individually weak effects.

Multiple classes of predictive models were trained for prediction of wound outcome, including penalized logistic regression (glmnet), support vector machine (SVM), neural network (NN), and random forest (RF). The flexibility of RF, NN, and SVM (radial kernel) models is expected to handle complex interactions. The penalized logistic regression is expected to find a balance between a small subset of features that have the strongest impact on outcome and features with individually large effects. Each model was trained using the boot632^24^ method to estimate performance for tuning.

Feature importance values were examined for each model class (Supplementary Figure S10). Feature importance rankings are model-dependent in each case, except for SVM, where feature importance is calculated from the data in a model independent way. Detection of *Pseudomonas putida* by whole metagenome sequencing was identified by all models as the most important nosocomial species feature for predicting wound failure (rank # 5, 4, 12 in glmnet, NN, and RF respectively; #6 model independent importance). Microbial diversity (as measured via genus Hill’s N_1_) was also identified as a top feature by all models (rank # 13, 14, 10; #15). Detection of *A. baumannii* by whole metagenome sequencing was predictive of outcome via model independent assessment (rank #17), but was not in the top 20 important features in glmnet, NN, or RF. Conversely, *E. faecium* was important only in RF (rank #13). Detection of genes conferring beta-lactam resistance by targeted sequencing was the top ranked AMR-associated feature prior to modeling and in glmnet and NN models (trimethoprim resistance was highest ranked in RF). AMR features were not, however, observed in the top 20 feature importance list in any model.

### Classification models trained with microbial features

The presence of metagenomic-derived microbial features in variable importance analyses indicate their predictive value. The features employed in these analyses also included clinical wound characteristics that associate with wound outcome. To assess the impact of adding metagenomic variables to a feature set containing only clinical variables, two feature sets were used for performance comparison, (1) a set containing only medical covariates and (2) a set including both medical covariates and targeted/whole metagenome sequencing-derived microbial variables. Performance for the four model classes trained with all clinical and microbial variables is given in Table 3. These metrics were directly compared to models trained with clinical covariates only (no microbial features). No statistically significant differences between model pairs trained with the two distinct feature sets were observed (Supplementary Figure S11).

**Table 3:** Training set performance of machine learning classifiers for prediction of wound outcome using all clinical and microbial metagenomic variables.

Model	Probability threshold (%)	Median (%)	Mean (%)	q1 (%)	q3 (%)
**Precision**					
rf	35.00	66.67	65.27	55.60	75.00
nnet	40.00	57.14	57.84	50.00	66.70
glmnet	45.00	60.00	59.85	50.00	66.70
svmRadial	20.00	66.67	64.45	50.00	75.00
**Sensitivity**					
rf	35.00	83.33	80.15	71.40	90.00
nnet	40.00	75.00	73.82	62.50	85.70
glmnet	45.00	71.43	71.94	62.50	83.30
svmRadial	20.00	66.67	66.76	50.00	83.30
**Specificity**					
rf	35.00	85.71	85.42	80.00	91.30
nnet	40.00	82.97	82.16	76.40	87.50
glmnet	45.00	84.62	83.93	78.90	90.00
svmRadial	20.00	88.24	86.58	81.20	95.00

Four distinct machine learning classifiers (rf = random forest; nnet = neural network; glmnet = penalized logistic regression; svmRadial = support vector machine) were applied to the training data set after training on identical features. These features were composed of wound characteristics, antimicrobial resistance detection variables, and nosocomial pathogen sequence detection. Summary statistics for held-out boot632 estimates of performance metrics for model performance are shown for each classifier at their optimal threshold for distinguishing wound outcomes (i.e., classification at the threshold which maximizes Youden’s J).

## Discussion

An improved understanding of combat wound bioburden features would aid treatment planning and intervention strategies. Microbial culture underestimates true bioburden^7,25,26^, and even when molecular methods are applied, measuring only a subset of microbes may not be sufficient. Comprehensive metagenomic analysis of microbial populations could inform health-relevant parameters^27,28^. Our study begins to address this issue through whole metagenome and targeted assessment of samples from a cohort of combat-injured patients.

Concordance analyses demonstrated agreement between quantitative bacteriology (QBAC) and metagenomic results, especially for high prevalence microbes, and metagenomics identified microbes not detected by bacteriology, indicating that metagenomic variables accurately and sensitively describe bioburden composition. Metagenomic detection depends on a range of factors, including genome size, relative abundance, reference database depth, and chosen thresholds. QBAC will inherently bias toward culturable microorganisms. The discordance observed could be due to variance in genomic parameters between genera and the presence of culturable genera below the metagenomic limit of detection.

The observed metagenomic features provide a portrait of combat wound bioburden and suggest that there is not a single microbial profile representative of wound outcome. Many categories of microbial and non-microbial observations exist, and many molecular and clinical factors, which may or may not operate coupled to microbial involvement, play roles in trauma outcome^29–32^. Detection of *Pseudomonas* via metagenomic sequencing did associate with wound failure. Colonization with *Pseudomonas* was documented in combat casualties evacuated to the U.S. during 2005–2009^33^, was prevalent in international studies of both military and civilian wounds^34^, and associates with wound failure via microarray-based detection^7^. These results add to an existing body of evidence that *Pseudomonas* exert detrimental effects on wound healing.

Our results identified correlated pairs of microbial genera, which may reflect that: (1) mutually beneficial interactions exist between microorganisms, (2) wound conditions are amenable to members of both genera, or (3) these genera are prevalent across subsets of wounds (e.g., group by geography, body site, patient). Further studies may clarify whether co-infection events are more virulent than single infections, as in chronic wounds with *S. aureus* and *P. aeruginosa*^35^. Assessment of multiple intermediate specimens for a single wound (though occurring in a minority of cases) does raise the possibility of co-linearity within a wound, i.e.*,* patients with higher sample numbers may be more heavily weighted. All intermediate specimens were included in the analysis to avoid excluding any data available from these unique cohorts and irreplaceable specimen sources; however, conclusions should be interpreted in this context.

Past studies suggest that both increases and decreases in microbiome diversity associate with poor outcomes^36^. The relationship of microbial diversity with disease is complex and varies according to individual and condition^37^. It is therefore not unexpected that significant associations between diversity metrics and wound outcome were not observed. The reduced diversity in samples from failed wounds may suggest an increasing risk of single taxon outgrowth under a dysregulated physiological response. More studies are necessary to clarify this finding. Reduced diversity at later time points may reflect a smaller number of taxa amenable to survival under selective hospital conditions.

Non-targeted metagenomic techniques allow increased resolution and broader scope^36^, but reduced coverage. We therefore applied a previously developed^16^ panel for targeted amplification of genomic AMR factors. AMR detection in this study references genome level detection of sequence associated with resistance and does not reflect phenotypic or transcriptomic susceptibility assessment. Confirmation of susceptibility phenotype would require further evaluation; however, this study aimed to assess a broad range of microbial metagenomic variables that have not previously been examined in the context of traumatic injury, and to integrate such observations in statistical models. Genome level evaluation allowed for comprehensive measurement of many AMR variables in parallel.

Logistic regression models revealed substantial associations (odds ratios > 1.5) between genomic resistance signatures and four ESKAPE pathogens (*A. baumannii, K. pneumoniae, P. aeruginosa, S. aureus*), suggesting these signatures are coincident with the indicated species. Five out of the top 12 predictors with odds ratios > 1.5 included *A. baumannii*, highlighting its potential for multidrug resistance in combat wounds. *A. baumannii*, a colonizer of traumatic injuries from combat environments that is persistent and difficult to treat^3,30,33^, associated significantly with resistance to antimicrobial classes including trimethoprim and aminoglycosides in this study’s data. Though further sequencing and analysis would be required to ascertain whether *A. baumannii* bacteria are carrying these resistance genes in these samples, the *A. baumannii* reference genome is known to harbor many classes of drug resistance genes including to diaminopyrimidines (trimethoprim class) and aminoglycosides. MDR isolates of *Acinetobacter* have shown > 70% resistance to trimethoprim/sulfamethoxazole (TMP-SMX), with extensively drug resistant (XDR) isolates exhibiting near complete resistance^38^. Aminoglycoside resistance is observed in approximately 50% of clinical isolates^39^, conferred by a range of microbial acetyl- and phosphotransferase genes. Despite its AMR profile, neither *A. baumannii*’s abundance nor presence of resistance gene classes were top predictive features in subsequent machine learning analyses. Treatment with drugs to which it may be resistant were among the top predictive features, however, highlighting the importance of managing AMR. Employing these data as guidance could reduce MDRO outgrowth, particularly when aminoglycosides are administered.

Elevated prevalence of AMR signatures was observed for wound outcome, MODS, and wound type. Dysregulated conditions may create an environment less likely to effectively contain colonization, thereby allowing expansion of resistant subpopulations. Deployed patients with MDR gram-negative bacilli infections do display a higher prevalence of traumatic amputation and higher injury severity scores^40^. This is also supported by organ failure observations, including cirrhosis^41^ and pancreatitis^42^, in which MDR infections associate with poor outcome. Association of AMR signatures with wound type (primarily STIs) supports observations that traumatic wound microbiomes reflect injury mechanism^43^.

Several of the strongest associations between AMR genes and antibiotic administration in failed wounds were within the class of beta-lactam resistance. The emergence and increasing prevalence of beta-lactam resistant microorganisms has been documented in many trauma applications including surgical site infections^44^ and burn injuries^45,46^. One of the gene factors identified in this study, *blaOXA-50*, was recently identified as one of the primary epidemiological characteristics of hospital-derived *P. aeruginosa* isolates^47^. Potential association of such genes with wound failure in this study could inform future selection of genomic determinants indicative of clinical outcomes.

Machine learning classifiers have been applied to combat injuries for prediction of pneumonia^19^, infection^48^, closure timing^29^, venous thromboembolism^18^, and heterotopic ossification^49^. The utility of clinical variables for predicting outcomes was demonstrated in these studies, thus the current study employed a microbe-centric focus. Our classification analysis aimed to determine (1) which individual metagenomic features exhibit relative predictive importance and (2) whether a more predictive model can be learned through addition of metagenomic features to medical covariates.

The most consistently predictive microbial variable across models was *P. putida*. *P. putida* infection in civilians is generally associated with immunocompromised patients^50^; however, it has been documented in wounds from combat injuries, which are known to exhibit uncommon infectious profiles^51^. These results reinforce the importance of examining service member populations when developing prognostic approaches for wounded warfighters. Microbial diversity was identified as an important predictor of wound outcome, suggesting that samples from wounds with lower microbial diversity have an increasing likelihood of being derived from failed wounds.

The injury patterns associated with wounds examined in this study are from counter-insurgency, asymmetric warfare. In future conflicts reflecting similar conditions, these results show the *potential* to identify up to 80% of wound failures from all given variables in the models from this study. Given the presumption that a wound predicted to fail would be redirected toward an alternative intervention, the false discovery rate (FDR) should also be considered, i.e.*,* the proportion of wounds that would receive alternative treatment based on the predictive indicator (denominator), but that would have healed with the current standard of care (numerator). For the decision threshold presented (maximizes sensitivity over a random classifier), a precision of 58–65% (dependent on model) implies that approximately 35–42% (where FDR = 1 – precision) of wounds receiving modified or personalized care would be expected to otherwise heal with the current diagnostic standard, i.e., serial debridement followed by primary closure in the absence of sequence-based microbial data. These estimates are made using the dataset available in this study; however, in practice, further adjustment could achieve more equitable distribution of resources and care.

The depth of sequence data was generally higher for effluent relative to tissue specimens, and sample type separation to account for differences further reduced the number of observations permissibly included. Despite sample quantity limitations, our results indicate that similar models trained on wound specimen data from a larger number of patients could play a critical role in calling out adverse outcomes, facilitating precision care without substantially increasing the burden of personalized treatment.

Our data did not indicate a statistical difference between the predictive performance of a model trained with or without metagenomic microbial variables. Although two microbial features were among the most important features (in the models trained with microbial features), it is possible that the flexibility of the classifiers allowed a different equally performant model to be learned from the non-microbial features alone. Larger training sets may also increase the power to detect subtle differences in predictive performance and feature importances. The utility of microbial data will also vary by patient. The inflammatory response of each individual to injury will vary based on both environmental and host-specific factors, including medical interventions that may mask cases where microbial metagenomic information would otherwise be predictive. As such, for individuals with minimal contamination, value of microbial metagenomic features may be limited. However, for patients with extensive pathogen-mediated inflammatory events, such information may be critically predictive, especially if not ascertainable by other methods. Further studies employing explicitly defined or explainable machine learning models will be required to assess the complex interplay between microbial metagenomic variables and variables representing the host immune response.

To avoid overrepresenting microbial variable impact, models did not employ metagenomic variables exclusively. The current analysis explicitly examined variation explained by metagenomic variables after accounting for wound variables (e.g., type, size, severity), anticipating that these metrics would be clinically available and can be expected to exert substantial effects. Although the latter analysis could provide additional information, our study aimed to assess enhancement of predictive capacity relative to the standard of care (currently available clinical features). Omission of clinical features would result in misleadingly high effects for microbial variables. For instance, in a scenario where the dominating wound feature is nearly exclusively size or severity, associations with genera or combinations of genera and resistance genes, while insightful, could be spurious. Therefore, additional studies are necessary to clarify the prognostic value of isolated metagenomic data.

In summary, our study demonstrates the application of statistical and machine learning methods to a rich set of metagenomic features and suggests that metagenomic data may supplement culture methods by monitoring wound bioburden and evaluating the association of microbial constituents with genomic signatures of antimicrobial resistance. These metagenomic features provide a detailed array of information with potential utility for informing future treatment of combat extremity wounds.

## Methods

### Sample collection and processing

Samples were previously collected from U.S. service member patients injured in Iraq and Afghanistan in compliance with all federal regulations governing the protection of human subjects and informed consent (Walter Reed National Military Medical Center Institutional Review Board protocol #352334).

Sample handling procedures were carried out as previously described^7,30,52^. Briefly, surgical debridements were performed every 48–72 h up until delayed wound closure. Tissue samples were obtained from the center of the wound. Wound effluent samples were composed of fluid from the wound site, obtained from the canisters of a negative-pressure wound therapy device. All samples were stored at −80 °C until analysis.

Samples were obtained at timepoints (washouts) corresponding to one of three categories: initial debridement (first assessment upon arrival at WRNMMC), intermediate (variable number of specimens collected between initial and final), and final debridement (last specimen collection prior to closure). Wound tissue biopsy and effluent samples from 78 wounds in 56 combat-injured patients were available for analysis. Of 78 total wounds, tissue biopsies were available for 73 wounds, and effluent available for 60 wounds. Both effluent and tissue samples were available for 55 wounds.

Samples represent wounds that either healed and resolved successfully or failed to heal. For the purposes of this study, wound failure was defined as one or more of the following incidents: frank dehiscence post-closure, necessity of reoperation for persistent drainage, progressive erythema, or < 90% engraftment of an applied split-thickness skin graft. Where outcome was not available, the corresponding wounds are indicated as such. Data from these wounds was not included in analyses of indicators for wound outcome.

### Sample extraction and purification

The current study was reviewed and approved by the Lawrence Livermore National Laboratory Institutional Review Board. All methods were performed according to relevant regulations, and experimental protocols were carried out in accordance with guidelines from relevant institutional committees.

Genomic DNA was extracted from samples using the cador pathogen kit (Qiagen). Tissue (10–30 mg) was digested with proteinase K and incubated at 56 °C. Resultant tissue lysate, or liquid effluent (200 µL), were mechanically lysed with buffer ATL and lysing matrix A using the Fast-Prep-24 (MP Biomedicals). Nucleic acid was purified from lysates using QIAamp mini columns according to the manufacturer’s standard protocol. Genomic DNA was quantified and quality assessed using the Qubit dsDNA HS kit and NanoDrop OneC (Thermo Fisher).

### Whole metagenome sequencing

Genomic DNA samples were prepared for whole metagenome sequencing using the Nextera DNA Flex library preparation kit (Illumina). Library quality was confirmed using the TapeStation 4200 (Agilent). Any gDNA samples generating low quality libraries were re-purified via ethanol extraction. Resultant libraries were sequenced on the NextSeq 500 using the 300-cycle high output kit (V2, Illumina).

### Metagenomic analysis and taxonomic classification

Sequence data were assigned to taxonomy using the Livermore Metagenomics Analysis Toolkit (LMAT), which is a metagenomic analysis pipeline that searches for taxonomic identifiers associated with *k*-mers found in a corresponding reference genome database^53,54^. In recent comparative studies, the LMAT platform demonstrated good limits of detection (~ 80% sensitivity for genomes with 0.04X coverage), and precision (20–100%)^55,56^. These parameters are tunable via thresholding on abundance. Per sample relative abundance corresponding to each taxonomic level was estimated from the proportion of total reads assigned on a per sample basis. Though a useful indicator of taxonomic abundance, these representations are biased according to genome lengths and copy numbers of the corresponding reference sequences.

Reads mapping to taxonomic identifiers at the genus and species level were binned according to genus and species, respectively, and counted. Downstream analyses treat counts as is or as relative abundances (i.e., as a ratio of reads assigned to a given taxa relative to another quantity, such as the total number of reads for a given sample, the average number of reads across taxa in a sample, or a predetermined taxon’s read count). Microbial-mapped content represented a low proportion of total sequence data due to human genomic background. While enrichment techniques have the potential to increase microbial sequence proportion, a non-targeted approach was maintained in this study to provide enhanced taxonomic resolution, interkingdom detection, and reduced bias from amplification and experimental manipulation.

### Comparative analysis of metagenomic sequencing with quantitative bacteriology

Quantitative bacteriology was previously performed as described^30^. The technique used to assess agreement between sequencing and microbiological techniques was drawn from U.S. Food and Drug Administration (FDA) guidance on reporting results from the evaluation of diagnostic tests when there is no gold standard^57^. In this circumstance, one test/method is designated as “new” and the other the “non-reference standard,” which serves as the comparator. While other methods of agreement, such as Cohen’s kappa and Scott’s pi have been employed in the past, recent research has found that these methods have limitations, including overcorrection for chance agreement and bias toward the underlying prevalence of the infection^58^. As such, current FDA guidance recommends calculating more straightforward measures of agreement: the positive percent agreement (PPA), negative percent agreement (NPA) and overall percent agreement (OPA).

Three measures of agreement were calculated using a 2 × 2 table format indicating prevalence of positive/negative results for both the “new test” (metagenomic sequencing) and “non-reference standard” (quantitative bacteriology). From these data, the positive, negative, and overall percent agreement were calculated. All measures used the non-reference standard as the denominator in calculations. For example, the positive percent agreement is the proportion of “non-reference standard” positives that are also “new test” positives (analogous to a sensitivity calculation). Two-sided 95% confidence intervals conditional on the observed non-reference standard results (ignoring the variability in a non-reference standard) were calculated using Wilson’s Score method with the binom R package^59^.

### Targeted amplification and sequencing of genomic resistance signatures

Targeted amplification of resistance-associated genomic signatures was performed using an Ion AmpliSeq panel (AmpliSeq MDR v.1, Thermo Fisher). This panel includes primer pools designed for generation of 1358 amplicons targeting 518 non-SNP mediated resistance-associated genes, and has been previously applied for detection of resistance signatures associated with the International Space Station^16^. A subset of 478 validated genes (815 amplicons) was analyzed for the purposes of this study. Libraries were prepared using the AmpliSeq protocol according to the manufacturer’s recommendations. Templating and sequencing were performed using the Ion Chef and Ion S5 (Thermo Fisher).

Titrated copy numbers of reference gDNA from *Acinetobacter baumannii* and *Pseudomonas aeruginosa*, spiked into human reference gDNA background, were employed as positive controls for calibration. An amplicon detection threshold of 100 reads was empirically selected for detection of a given amplicon, and a gene was declared present if any corresponding amplicon was detected. No AMR genes were detected in two human-only reference samples when using amplicon thresholds of 53 and 76 reads (Mean + 2SD = 97). Certain genes were “tiled” by primers/amplicons spanning the full breadth of the gene, to support non-ambiguous detection in cases where genes conserved sequence with non-AMR associated genes. For these genes, a stricter threshold of 50% positive amplicon detection events was selected. These thresholds were empirically assigned to minimize false positive detection events in background while achieving acceptable sensitivity.

### Statistical processing and analyses

#### Heatmap visualization

Heatmaps were constructed and hierarchical clustering performed with Euclidean distance via the complete linkage method, using the pheatmap R package^60^.

#### Sample ordination

Samples, which are high-dimensional compositional vectors of genus counts, were represented in a lower dimensional space by performing Principal Coordinates Analysis (PCoA). No genera were removed, i.e., the sample-sample distances account for non-microbial and unknown genera. Because PCoA assumes Euclidean distances, and the samples exist in the simplex, the fraction of reads for each genus in each sample were transformed from the simplex to Euclidean space with the isometric log transform (ILR). A pseudo-count of 1 read was added to each genus to avoid taking the log of zero. Transformation, ordination and visualization was carried out with the Compositional^61^, phyloseq^62^, vegan^63^, and ggplot2^64^ R packages.

#### Genus association analyses

Association tests were performed to examine the co-incidence of all pairs of microbial genera detected above a relative abundance threshold of 1 × 10^−4^, using Fisher’s exact test (R function `fisher.test`), and asymptotic Chi-squared (R function `chisq.test`) and G-tests (in-house script). 2 × 2 contingency tables were constructed for each time point (initial, intermediate, final) and for each pair of genera, where cell counts are the number of samples with either, none, or both genera present. Each given genus was indicated as present if its corresponding sequence read relative abundance was > 1 × 10^−4^. Pseudocounts were employed to avoid inconvenient parsing of cases with zero counts. This also has the effect of incorporating a prior belief that there are no associations. Furthermore, the G-test was performed with cell frequency estimates shrunk towards uniformity to account for biases and small sample sizes (`entropy::mi.shrink`)^65^. Results were reported for Fisher’s exact test *P* < 0.01. Fisher P values were adjusted to control the false discovery rate within each timepoint.

#### Alpha diversity quantification

Diversity was quantified at the genus level using the Hill numbers N_0_, N_1_, and N_2_, which represent the effective number of genera present^66^, weighted by proportion of metagenomic DNA sequences corresponding to each given taxon per sample.

#### Sample prevalence calculation

We call sample prevalence the proportion of samples in which a genus was assigned reads at a relative abundance exceeding 1 × 10^−5^. Sample prevalence was calculated out of samples for each time point (initial, intermediate, final), for each sample type (tissue, effluent), and for each final outcome of the wound (healed, failed). The R function `fisher.test` (Fisher’s exact test) was used to test whether the sample prevalence was significantly different between healed and failed wounds. For 2 × 2 tables, the odds ratio (prevalence in failed vs. healed) and 95% confidence interval are estimated.

#### Drug resistance gene class association analyses

Association tests were performed to identify drug resistance gene classes associated with different types of wounds (e.g., healed/failed, amputation/open fracture/STI). The R functions `prop.test` and `chisq.test` were used to test for each type of wound, type of sample, and time point. As previously noted, pseudocounts were added to all contingency tables to avoid inconvenient parsing of cases with zero counts and to encode a prior belief that there are no associations.

#### Nosocomial pathogen assessment

The `bayesglm` function of the `arm` package was used to fit logistic regression models for each antimicrobial resistance gene class. The model terms were a) the sample type (tissue, effluent) and b) log_2_ transformed read counts for each species (genus for *Enterobacter*). A pseudocount was added to all counts prior to performing the log_2_ transformation to avoid inclusion of zeroes. For purposes of this analysis both *P. aeruginosa* and *P. putida* were included.

#### Correlation assessment in antimicrobial gene and treatment class

Hierarchical All-against-All association (HAllA) was used for testing associations between high-dimensional heterogeneous datasets^67^. HAllA testing was applied to antimicrobial resistance gene detection and antimicrobial treatment regimen features. Samples were sorted into two groups according to the overall wound outcome (i.e., healed versus failed). To prevent the pre-analysis removal of highly prescribed antimicrobial treatment classes, such as beta-lactams, the entropy cut-off threshold for genes and treatment classes was reduced to 0.15 (--entropy 0.15). Normalized mutual information (NMI) was used to generate a similarity matrix (--metric nmi) among the AMR gene detection and treatment regimen features, and the resultant similarity matrices were used to create hierarchical clusters of features. Correlations (NMI) between the AMR gene detection and treatment regimen feature clusters were calculated between the cluster medoids. Statistical significance was determined by permutation testing, and P values were adjusted using the Benjamini–Hochberg method. Cluster correlations with Q < 0.05 were considered significant.

#### Machine learning classification

Sample exclusion: Only final specimens were employed for model training. Specimens with “NA” response label were excluded, resulting in 92 specimens. Feature selection: Feature categories included clinical covariates (*e.g.,* APACHE II score, wound size) and microbial features: targeted sequencing-derived AMR presence-absence (e.g.*,* tetracycline resistance gene present/absent) and shotgun metagenomic sequencing derived features (log read counts assigned to nosocomial pathogens, genus diversity metrics). Non-relevant features (antibiotic resistance categories with indeterminate drug assignments), features that encode the response label, features with high pairwise and average correlation, and zero-variance features were dropped. Linear polynomial effects of ordinal variables were kept while quadratic and cubic effects were discarded. Categorical variables were dummy encoded. Machine learning was performed with the `caret`^68^ package in R^69–73^. The models (random forests [rf]^74^, penalized logistic regression [glmnet]^75^, support vector machine [svmRadial]^76^, neural network [nnet]^77^) were automatically tuned to area under ROC curve (auROC) using the boot632 method^24^, i.e., averaging auROC calculated in resampled and unsampled samples, weighted by the probability of sampling an observation. Final bootstrap distributions of performance metrics for the best tuned models at optimal thresholds were calculated from only the “held out” (i.e., unsampled) samples from resampling iterations. Because models were trained at the sample level, predictions at the optimal *sample-wise* threshold for each model class (maximum Youden’s J) were summarized by wound to calculate performance at the wound level as follows: “failed” if any sample is predicted as “failed”, and “healed” if both samples are predicted as “healed”. This may hide false negatives resulting from tissue specimens with lower DNA yields, and likewise increase false positives from effluent specimens, which generated higher DNA yields. This was mitigated by including specimen type as a feature.

#### Model performance estimation

For comparison of models trained on two feature sets, estimates of performance were calculated on the unsampled samples from the bootstrap, which provided quantiles for the bootstrap distribution of performance metrics. Models were trained on sample-level observations; however, samples from the same wound are assigned the same outcome by definition. Sensitivity, specificity, and precision in predicting outcome were thus evaluated at the wound level.

## Supplementary Information

Supplementary Information 1.

Supplementary Information 2.

## Data Availability

The raw datasets generated during and analyzed in the current study are not publicly available due to sensitivities regarding their generation from injured military service member cohorts, but are available from the authors on reasonable request and in accordance with applicable regulations and data usage agreements.
