# Supplementary material for: Metagenomic features of bioburden serve as outcome indicators in combat extremity wounds
Source: Sci Rep. 2022 Aug 15;12:13816. doi: 10.1038/s41598-022-16170-x (PMC9378645; doi:10.1038/s41598-022-16170-x)
Supplement: Supplementary file 1 — Supplementary Information 1. [file 41598_2022_16170_MOESM1_ESM.pdf]

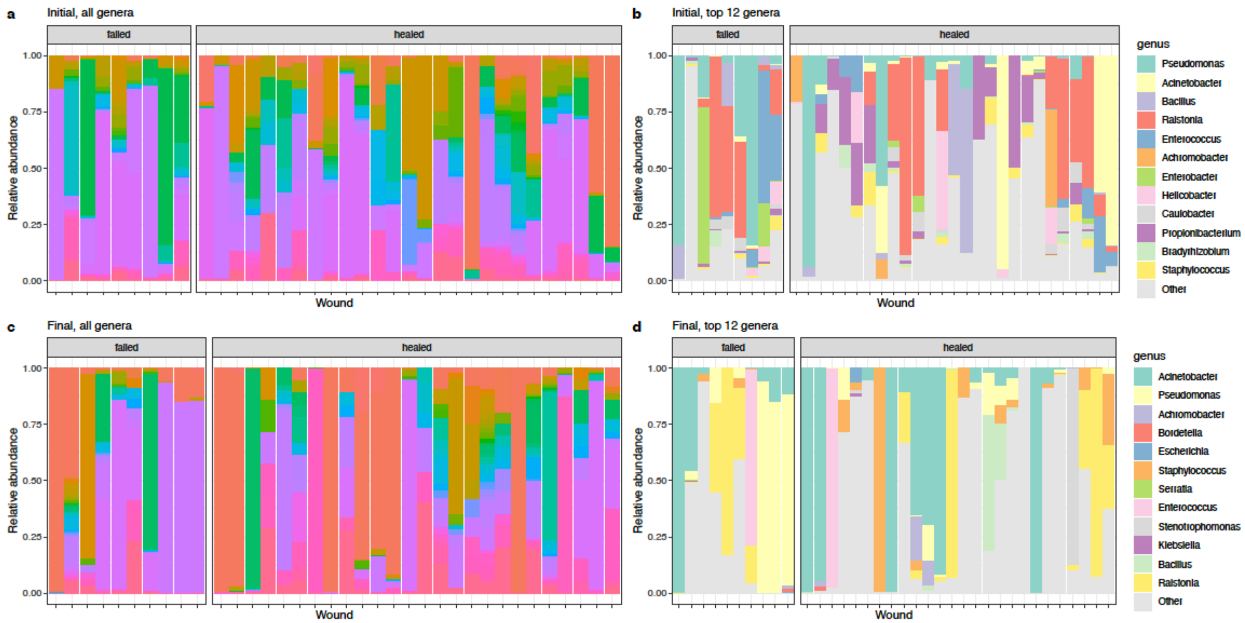


**Supplementary Figure S1. Relative abundance of sequence corresponding to microbial taxa.** Shotgun metagenomic sequencing and taxonomic classification of sequence was performed to examine microbial abundance profiles. Sequence read abundance obtained from wound effluent samples is shown per sample, relative to non-human total sequence, i.e., human-derived background sequence data was not included for calculation of abundance in this analysis. Panels indicate whether the specimen was obtained from a wound that healed successfully or failed to heal. Read abundance is shown for **A.** initial wound effluent specimens, showing all detected genera (filled colors indicate genus; legend not shown, as large number of genera cannot be visually represented); **B.** initial wound effluent specimens, with legend indicating the top 12 genera in legend colors; **C.** final wound effluent specimens, showing all detected genera (filled colors indicate genus; legend not shown, as large number of genera cannot be visually represented); **D.** final wound effluent specimens, with legend indicating the top 12 genera in legend colors. Legends provided are specific to each given panel.

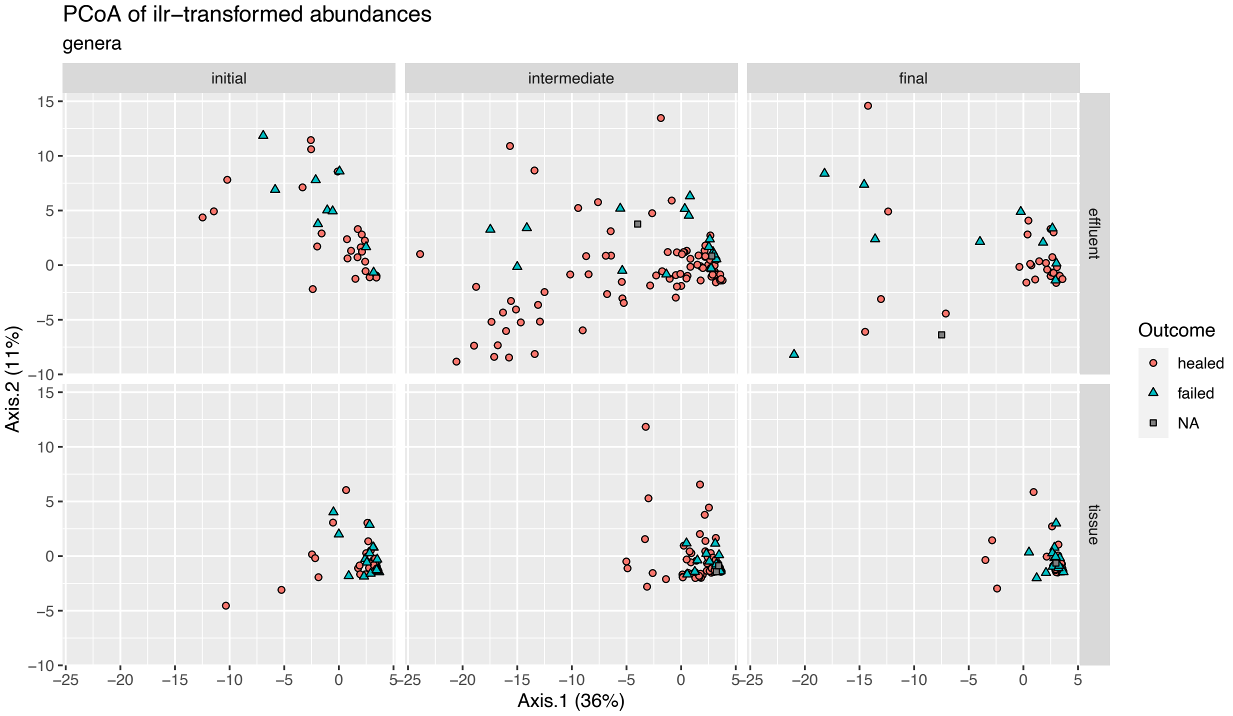


**Supplementary Figure S2. PCoA ordination of samples in low-dimensional space.** For each sample, reads were taxonomically classified at the genus level by LMAT. The fraction of reads for each genus in each sample were transformed from the simplex to Euclidean space with the isometric log transform (ILR). No genera were removed, and a pseudo-count of 1 read was added to each genus to avoid taking the log of zero. Principal coordinates analysis (PCoA) was applied to obtain a low dimensional representation of the samples. Percent variation captured by the first two dimensions are shown in parentheses. Samples are colored and shaped by outcome and displayed in separate panels by washout and sample type. NA: wound outcome not available.

| **Timepoint** | **Genus 1** | **Genus 2** | **Odds Ratio** | **95% CI** | **Fisher P value** | **Fisher Q value (BH)** |
|---|---|---|---|---|---|---|
| **intermediate** | *Acinetobacter* | *Escherichia* | 2.37E+01 | 3.03E+00–1.08E+03 | 1.59E-04 | 8.55E-02 |
| **intermediate** | *Escherichia* | *Serratia* | 5.48E+01 | 4.59E+00–2.98E+03 | 2.26E-04 | 8.55E-02 |
| **intermediate** | *Enterococcus* | *Streptococcus* | 1.32E+02 | 4.37E+00–1.03E+04 | 1.70E-03 | 8.55E-02 |
| **intermediate** | *Staphylococcus* | *Streptococcus* | 7.66E+01 | 3.02E+00–5.49E+03 | 3.38E-03 | 8.55E-02 |
| **intermediate** | *Enterococcus* | *Staphylococcus* | 7.66E+01 | 3.02E+00–5.49E+03 | 3.38E-03 | 8.55E-02 |
| **intermediate** | *Achromobacter* | *Bacillus* | 5.42E+01 | 2.30E+00–3.71E+03 | 5.60E-03 | 8.55E-02 |
| **intermediate** | *Bacillus* | *Citrobacter* | 5.42E+01 | 2.30E+00–3.71E+03 | 5.60E-03 | 8.55E-02 |
| **final** | *Achromobacter* | *Bordetella* | 6.81E+01 | 3.21E+00–5.31E+03 | 1.59E-03 | 1.50E-01 |
| **final** | *Acinetobacter* | *Pseudomonas* | 2.09E+01 | 1.89E+00–1.13E+03 | 3.61E-03 | 1.50E-01 |

**Supplementary Table S1. Statistical association of sequencing-based microbial genus detection.** Microbial genera were identified as present or absent at a relative sequence read abundance threshold of > 1 x $10^{-4}$. Pairwise analyses were performed for initial, intermediate, and final wound specimens. Results are shown at a significance level of Fisher's exact test P < 0.01. BH = Benjamini-Hochberg correction.

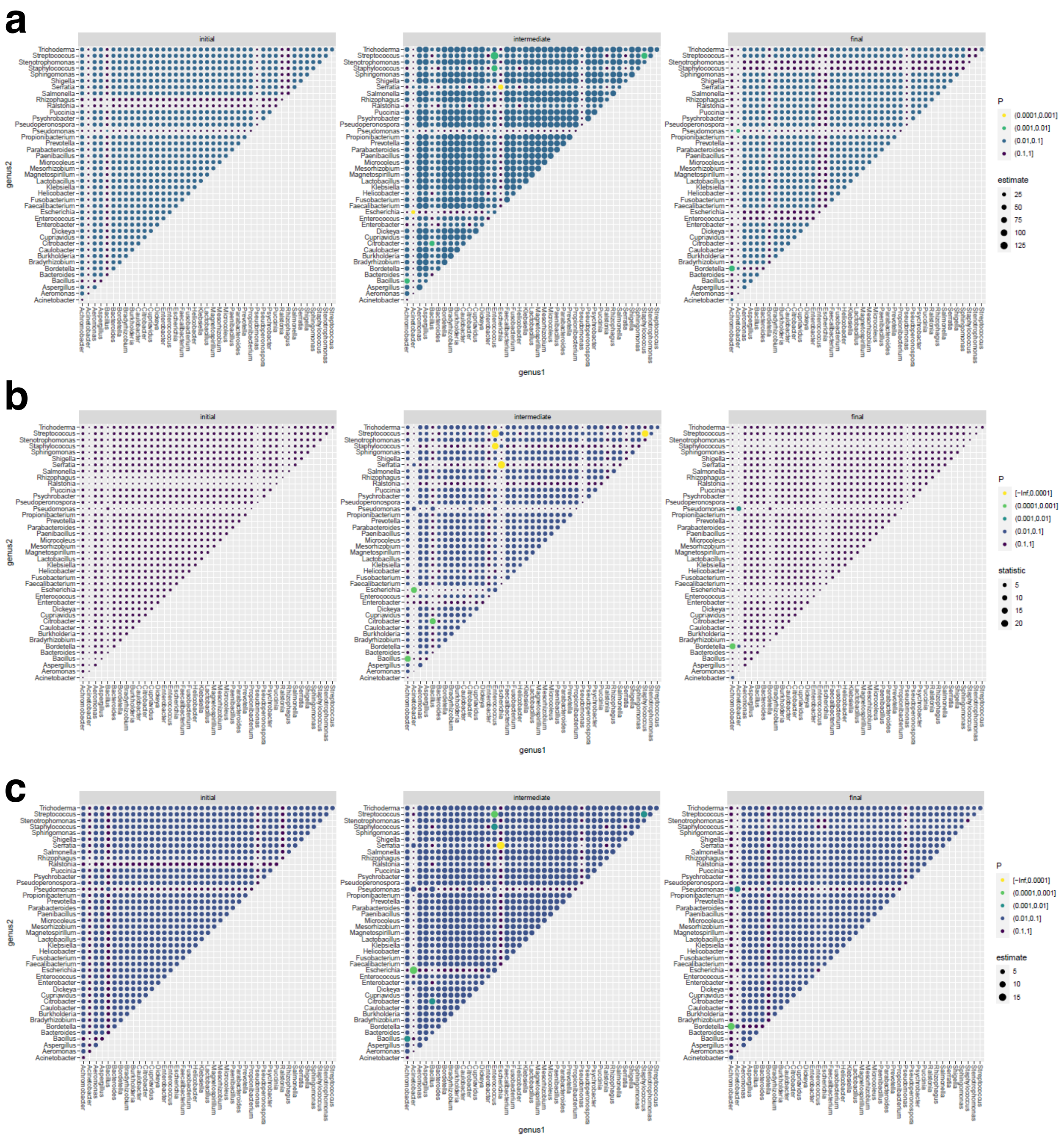


**Supplementary Figure S3. Correlograms indicating statistical association of microbial genus detection in wound effluent samples.** Microbial genera were identified as present or absent at a relative sequence read abundance threshold of > 1E-4. Pairwise analyses were performed for effluent samples within initial, intermediate, and final wound specimen categories. Three statistical tests were applied: **A.** Fisher's Exact Test, **B.** Chi-square test, and **C.** G-test.

| Species | QuantBAC Prevalence | Microbiome Percent Agreement | | |
|---|---|---|---|---|
| | | Positive | Negative | Overall |
| **Overall** | **57/66 (86.4%)** | **45/57 (78.9%) [66.7% - 87.5%]** | **6/9 (66.7%) [35.4% - 87.9%]** | **51/66 (77.3%) [65.8% - 85.7%]** |
| *Acinetobacter baumannii* | 36/66 (54.5%) | 34/36 (94.4%) [81.9% - 98.5%] | 0/0 | 34/36 (94.4%) [81.9% - 98.5%] |
| *Enterococcus faecium* | 10/66 (15.2%) | 4/10 (40%) [16.8% - 68.7%] | 0/0 | 4/10 (40%) [16.8% - 68.7%] |
| *Bacillus cereus* | 3/66 (4.5%) | 3/3 (100%) [43.9% - 100%] | 0/0 | 3/3 (100%) [43.9% - 100%] |
| *Enterococcus faecalis* | 3/66 (4.5%) | 0/3 (0%) [0% - 56.1%] | 0/0 | 0/3 (0%) [0% - 56.1%] |
| *Pseudomonas stutzeri* | 2/66 (3%) | 2/2 (100%) [34.2% - 100%] | 0/0 | 2/2 (100%) [34.2% - 100%] |
| *Escherichia coli* | 1/66 (1.5%) | 1/1 (100%) [20.7% - 100%] | 0/0 | 1/1 (100%) [20.7% - 100%] |
| *Staphylococcus epidermidis* | 1/66 (1.5%) | 1/1 (100%) [20.7% - 100%] | 0/0 | 1/1 (100%) [20.7% - 100%] |
| *Citrobacter freundii* | 1/66 (1.5%) | 0/1 (0%) [0% - 79.3%] | 0/0 | 0/1 (0%) [0% - 79.3%] |

**Supplementary Table S2. Concordance between microbial species-level detection by quantitative bacteriology and metagenomic sequencing.** Concordance was assessed according to prevalence, with percent agreement estimates used to compare genus detection from metagenomic sequencing to quantitative bacteriology, applied here as a non-reference standard.

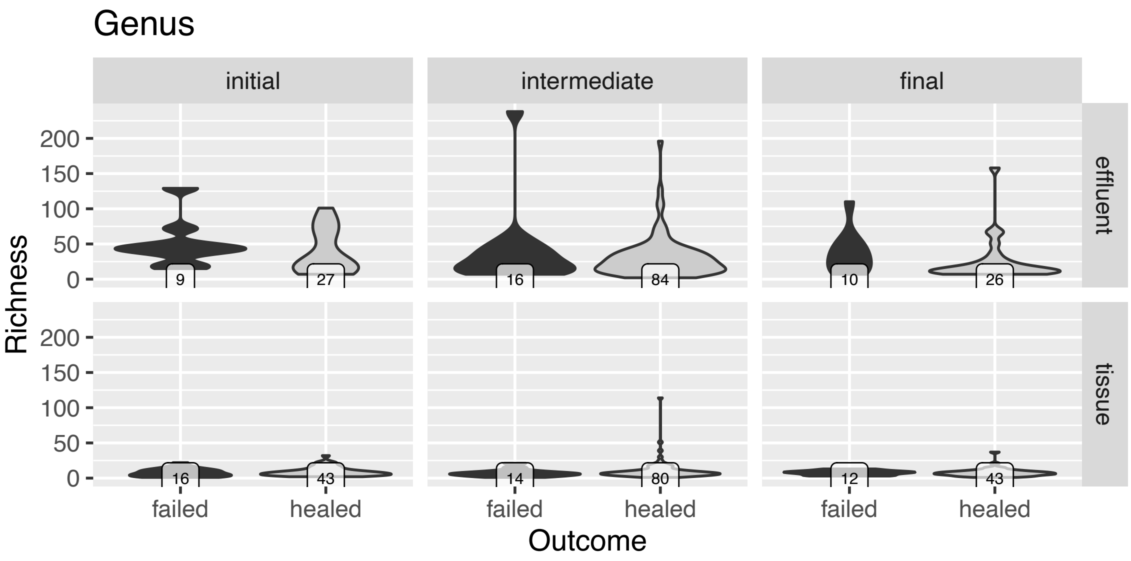


**Supplementary Figure S4. Genus richness distributions for groups of samples.** Violin plots show the density of samples with corresponding number of genera (richness). Violin plots are scaled to have the same area. Number of samples in each group are shown in box.

| Outcome | Washout | Type | Mean | SD | p0 | p25 | p50 | p75 | p100 | Hist | Num. Samples |
|---|---|---|---|---|---|---|---|---|---|---|---|
| **failed** | initial | effluent | 50.33 | 33.74 | 15 | 37.00 | 43.0 | 49.00 | 129 | 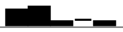 | 9 |
| **failed** | initial | tissue | 7.81 | 5.84 | 1 | 3.00 | 6.5 | 11.25 | 22 | 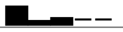 | 16 |
| **failed** | intermediate | effluent | 39.56 | 55.39 | 7 | 14.00 | 25.5 | 40.00 | 238 | 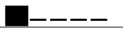 | 16 |
| **failed** | intermediate | tissue | 7.43 | 5.36 | 1 | 4.25 | 6.5 | 8.75 | 21 | 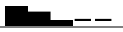 | 14 |
| **failed** | final | effluent | 36.70 | 30.75 | 7 | 14.00 | 33.5 | 48.00 | 110 | 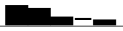 | 10 |
| **failed** | final | tissue | 7.58 | 3.29 | 3 | 5.50 | 8.0 | 9.25 | 13 | 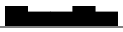 | 12 |
| **healed** | initial | effluent | 37.59 | 31.03 | 7 | 14.00 | 22.0 | 64.00 | 101 | 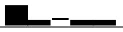 | 27 |
| **healed** | initial | tissue | 9.33 | 6.54 | 2 | 5.00 | 7.0 | 11.50 | 32 | 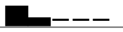 | 43 |
| **healed** | intermediate | effluent | 31.08 | 29.80 | 2 | 12.00 | 25.5 | 39.00 | 196 | 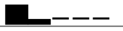 | 84 |
| **healed** | intermediate | tissue | 10.82 | 14.23 | 1 | 5.00 | 7.0 | 11.00 | 114 | 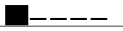 | 80 |
| **healed** | final | effluent | 25.50 | 31.97 | 7 | 10.00 | 14.0 | 24.75 | 158 | 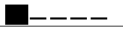 | 26 |
| **healed** | final | tissue | 7.95 | 6.08 | 1 | 5.00 | 7.0 | 9.00 | 37 | 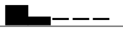 | 43 |

**Supplementary Table S3. Genus richness summaries for groups of samples.** Provided are the richness mean, sample standard deviation (SD), percentiles (pX), and a histogram (min = p0, max = p100, 5 evenly spaced bins). The number of specimens in each group are also shown.

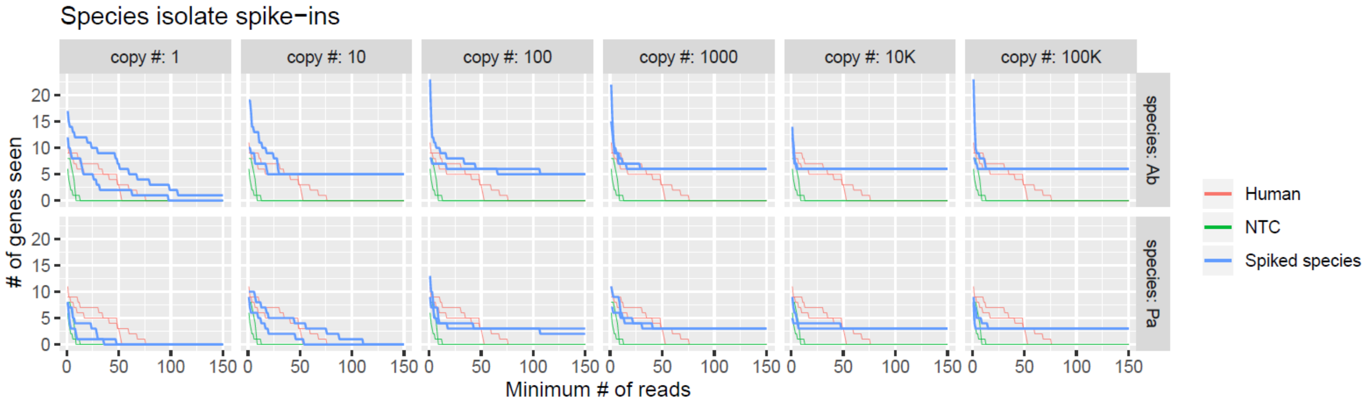


**Supplementary Figure S5. Impact of read count thresholds on detection of AMR genes via targeted sequencing.** Reference gDNA for *A. baumannii* and *P. aeruginosa* were spiked into a reference human background and subjected to targeted amplification and sequencing. Sequence read thresholds from 1-150 reads were tested to assess impact on number of detected genes. Results were subsequently employed toward selection of an operational threshold of 100 sequence reads. NTC = no template control.

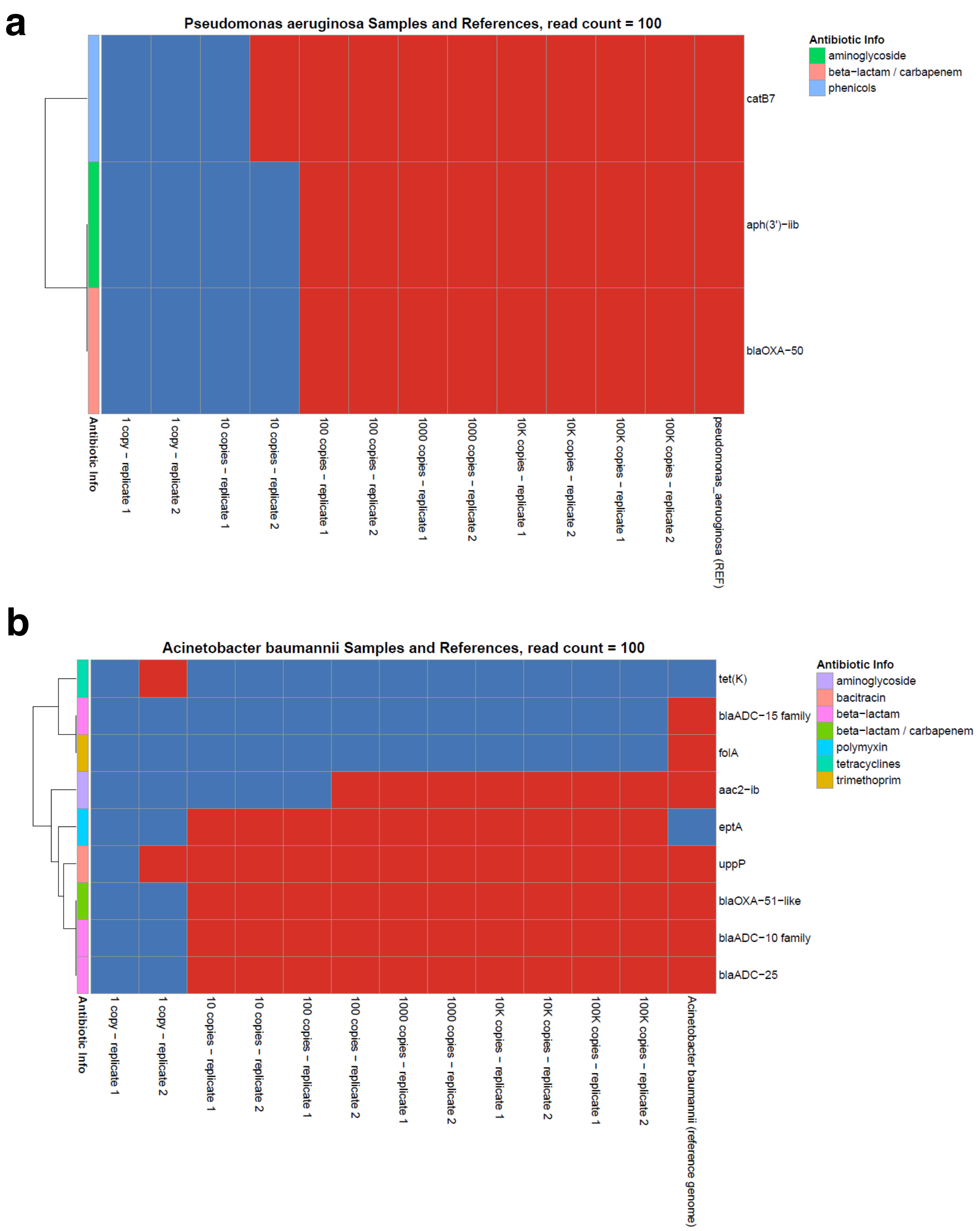


**Supplementary Figure S6. AMR gene detection from control reference species using targeted amplification.** A sequence read count threshold of 100 reads or greater was empirically

selected for detection of a given targeted amplicon, and an amplicon threshold of at least 50% of amplicons, was assigned for detection of a given gene. Gene presence for each represented gene was determined in reference sequences for strains of *Acinetobacter baumannii* and *Pseudomonas aeruginosa*. Genomic DNA corresponding to these strains was subjected to targeted sequencing and analysis at these thresholds. Detection results at titrated copy numbers for each species were compared to gene presence in the corresponding reference sequence for **A.** *Pseudomonas aeruginosa* and **B.** *Acinetobacter baumannii*.

| response | null.deviance | df.null | logLik | AIC | BIC | deviance | df.residual | p | padj |
|---|---|---|---|---|---|---|---|---|---|
| amr_Trimethoprim | 377.70 | 386 | -74.69 | 167.39 | 203.01 | 149.39 | 378 | 0.00 | 0.00 |
| amr_NA | 404.92 | 386 | -89.49 | 196.97 | 232.60 | 178.97 | 378 | 0.00 | 0.00 |
| amr_Aminoglycoside | 323.51 | 386 | -50.71 | 119.42 | 155.05 | 101.42 | 378 | 0.00 | 0.00 |
| amr_Bacitracin | 286.22 | 386 | -36.75 | 91.50 | 127.12 | 73.50 | 378 | 0.00 | 0.00 |
| amr_Polymyxin | 261.57 | 386 | -45.76 | 109.53 | 145.16 | 91.53 | 378 | 0.00 | 0.00 |
| amr_Tetracycline | 333.87 | 386 | -108.47 | 234.94 | 270.56 | 216.94 | 378 | 0.00 | 0.00 |
| amr_Beta-lactam | 350.34 | 386 | -130.49 | 278.98 | 314.61 | 260.98 | 378 | 0.00 | 0.00 |
| amr_Chloramphenicol | 92.86 | 386 | -25.02 | 68.05 | 103.67 | 50.05 | 378 | 0.00 | 0.00 |
| amr_Streptogramin | 53.42 | 386 | -9.58 | 37.16 | 72.79 | 19.16 | 378 | 0.00 | 0.00 |
| amr_Streptothricin | 92.86 | 386 | -30.97 | 79.94 | 115.57 | 61.94 | 378 | 0.00 | 0.00 |
| amr_Macrolide | 139.50 | 386 | -56.76 | 131.51 | 167.14 | 113.51 | 378 | 0.00 | 0.00 |
| amr_Fosfomycin | 25.05 | 386 | -1.91 | 21.82 | 57.44 | 3.82 | 378 | 0.01 | 0.01 |

**Supplementary Table S4. Logistic regression models for AMR genomic signature classes.** A logistic regression model was fit to the presence or absence of resistance categories detected by targeted sequencing. Models for 12 of the 18 classes represented by detected AMR resistance genes successfully generated models for predicting the presence of defined pathogens known to be associated with hospital-acquired infections.

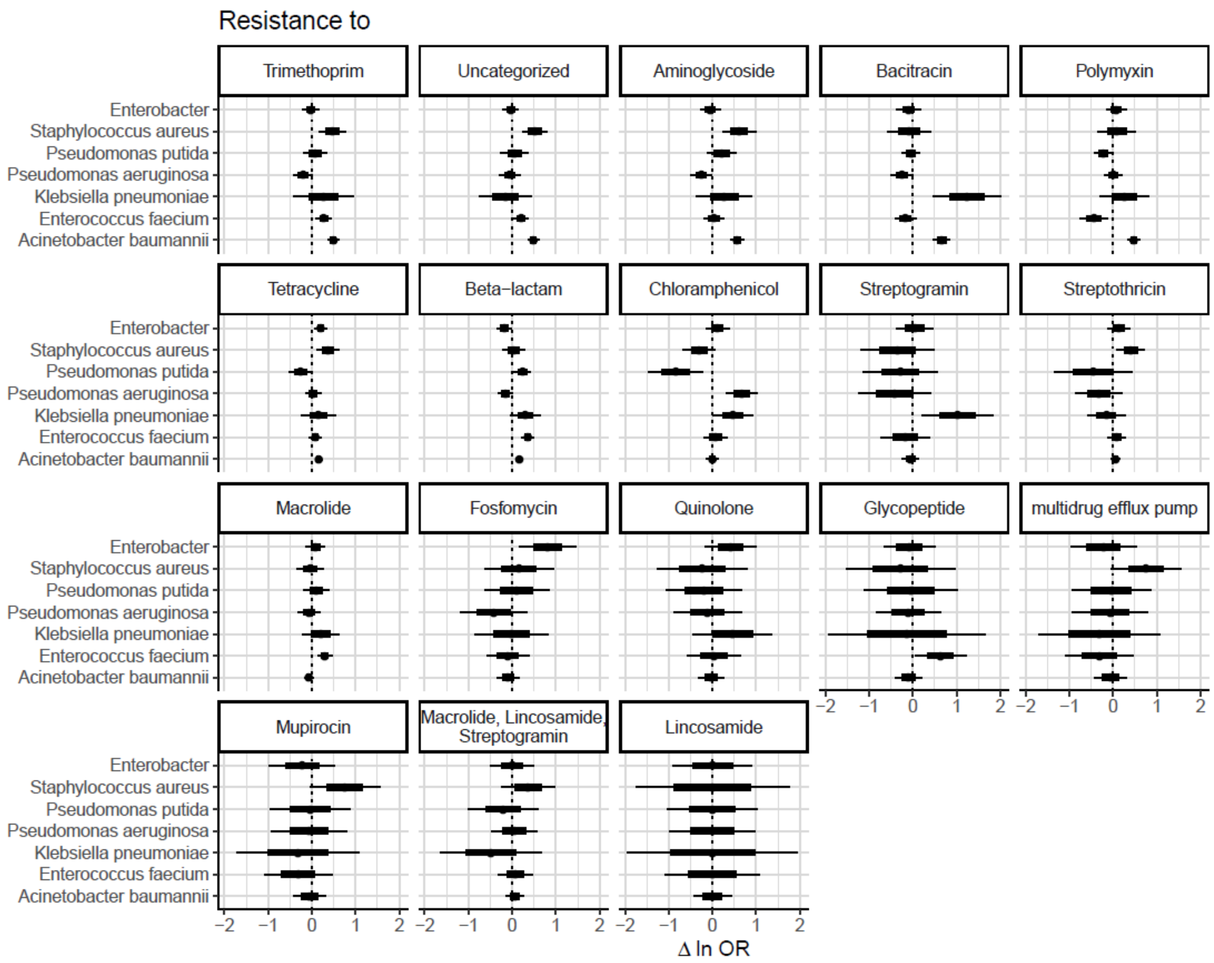


**Supplementary Figure S7. Effect sizes for associations between nosocomial pathogen detection and antimicrobial resistance signature category.** Logistic regression models were built to determine whether detection of defined genomic resistance signatures associated with metagenomic detection of specific genera corresponding to common nosocomial pathogens. Effect sizes for each examined microbial genus or species are shown along the vertical axis. Each category of antimicrobial resistance is shown in an individual panel.

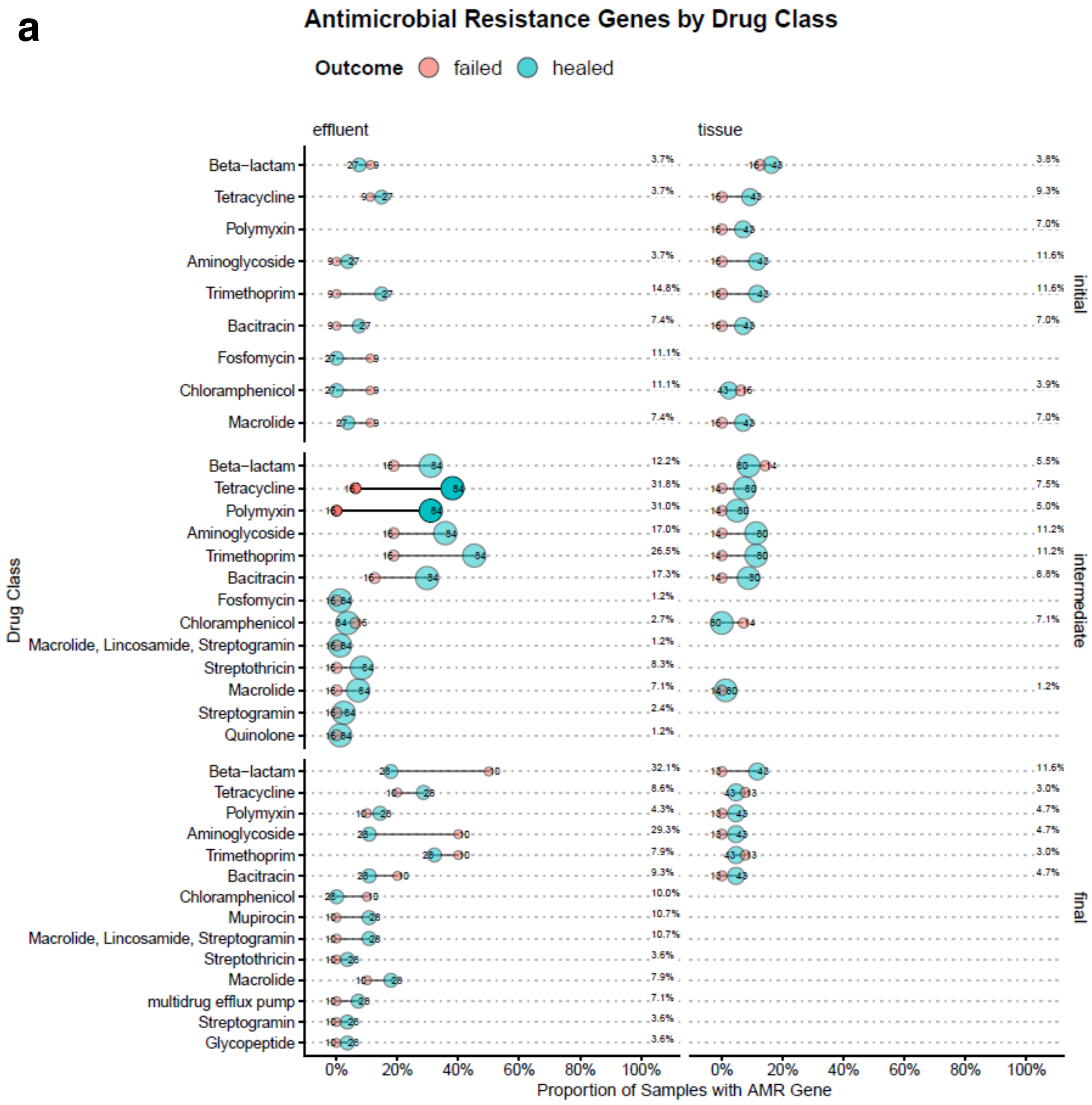
a
Antimicrobial Resistance Genes by Drug Class
Outcome failed healed
effluent
tissue
initial
intermediate
final
Drug Class
Beta−lactam
Tetracycline
Polymyxin
Aminoglycoside
Trimethoprim
Bacitracin
Fosfomycin
Chloramphenicol
Macrolide
Macrolide, Lincosamide, Streptogramin
Streptothricin
Streptogramin
Quinolone
Mupirocin
multidrug efflux pump
Glycopeptide
0% 20% 40% 60% 80% 100%
Proportion of Samples with AMR Gene

b

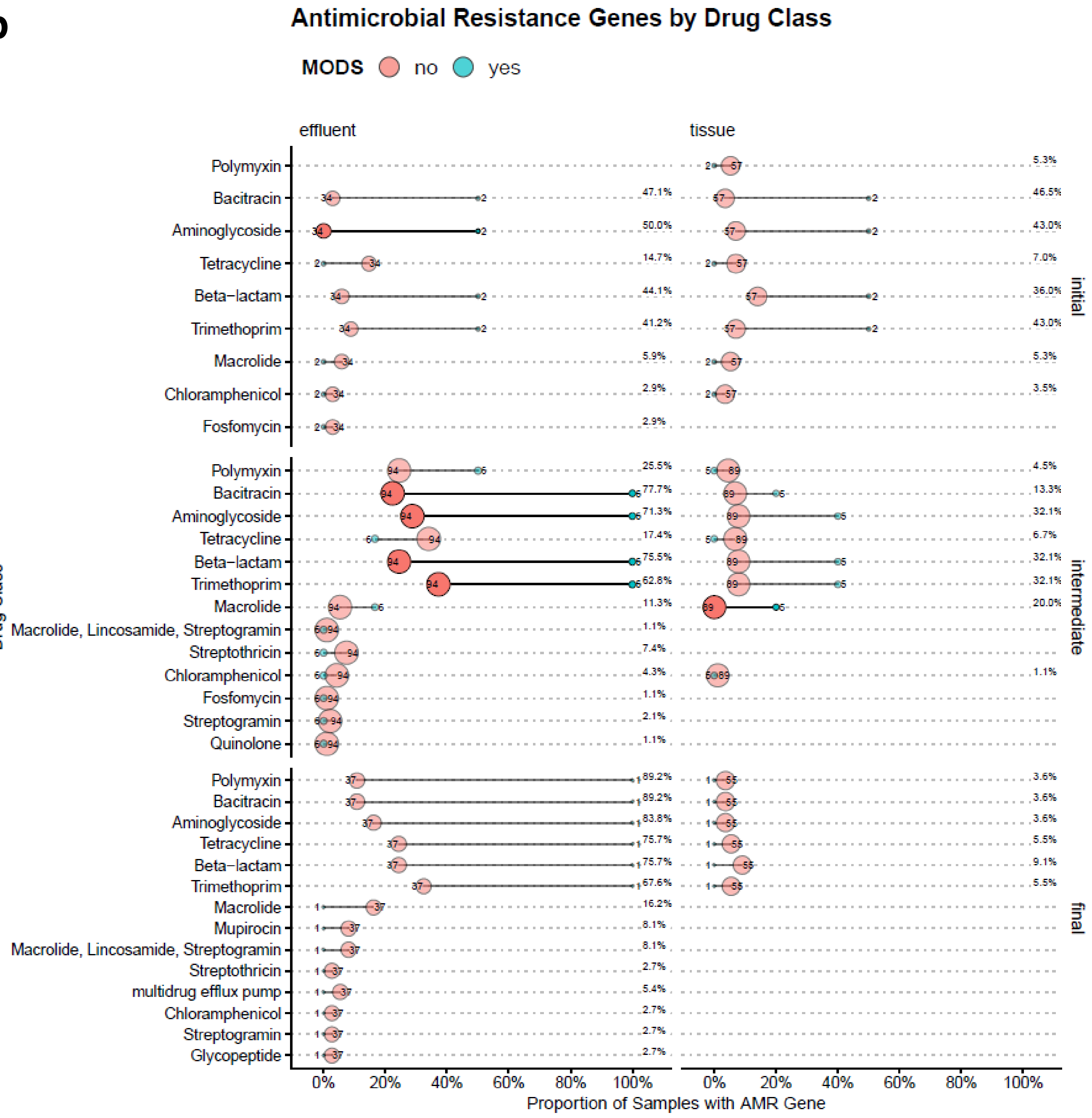
Antimicrobial Resistance Genes by Drug Class
MODS
no
yes
effluent
tissue
initial
intermediate
final
Drug Class
Polymyxin
Bacitracin
Aminoglycoside
Tetracycline
Beta−lactam
Trimethoprim
Macrolide
Chloramphenicol
Fosfomycin
Macrolide, Lincosamide, Streptogramin
Streptothricin
Streptogramin
Quinolone
Mupirocin
multidrug efflux pump
Glycopeptide
0%
20%
40%
60%
80%
100%
Proportion of Samples with AMR Gene

c

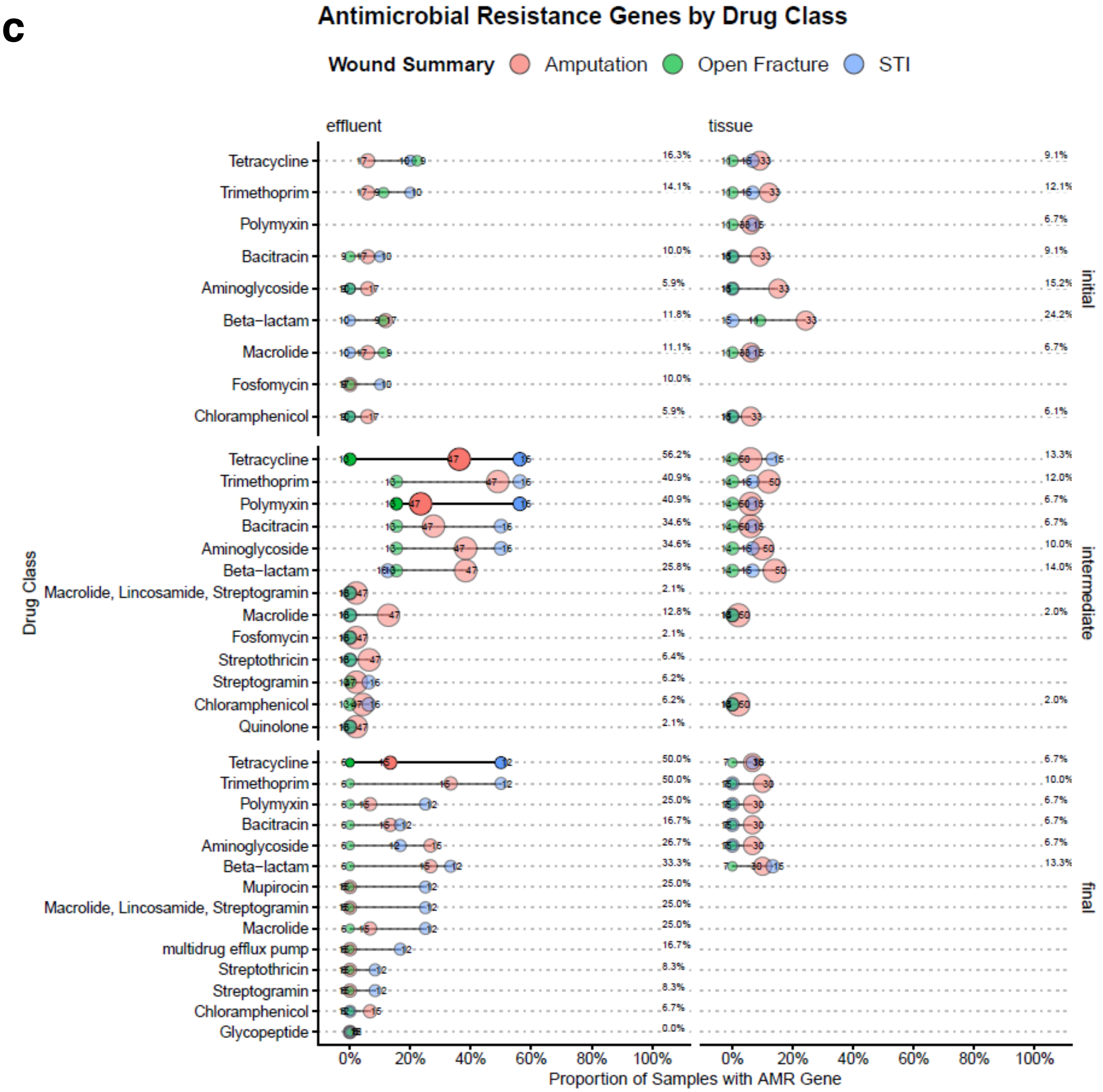

Antimicrobial Resistance Genes by Drug Class
Wound Summary
Amputation
Open Fracture
STI
effluent
tissue
initial
intermediate
final
Drug Class
Tetracycline
Trimethoprim
Polymyxin
Bacitracin
Aminoglycoside
Beta−lactam
Macrolide
Fosfomycin
Chloramphenicol
Macrolide, Lincosamide, Streptogramin
Streptothricin
Streptogramin
Quinolone
Mupirocin
multidrug efflux pump
Glycopeptide
0%
20%
40%
60%
80%
100%
Proportion of Samples with AMR Gene

**Supplementary Figure S8. Detection of AMR genes in clinically distinct wound subgroups.** Prevalence of antimicrobial resistance signature detection was calculated for wounds with distinct clinical parameters. Panels at left indicate effluent samples and panels at right indicate tissue samples. Top, middle, and bottom rows of panels indicate initial, intermediate, and final samplings respectively. As in previous analyses, prevalence is shown as a proportion of total healed or failed samples for each given sample category. Number of samples corresponding to each observation is given in text adjacent to the corresponding data point. The range between prevalence for each set of observations (i.e., difference in prevalence between wound category with highest prevalence for a given resistance class versus lowest prevalence for that class) is given in text to the right of each observation. Individually significant ($P < 0.05$) distinctions are shown with a bolded edge. No associations at $Q < 0.1$ are shown in this analysis, i.e., no distinctions were made at a threshold controlling the false positive rate below 10%. **A.** Genomic resistance detection prevalence in effluent and tissue specimens from wounds that either healed successfully or failed to heal. **B.** Genomic resistance detection prevalence in initial effluent and tissue specimens from wounds in patients with or without a diagnosis of multiple organ dysfunction syndrome (MODS). **C.** Genomic resistance detection prevalence in initial effluent and tissue specimens from distinct wound types (amputation, open fracture, soft tissue injury [STI]).

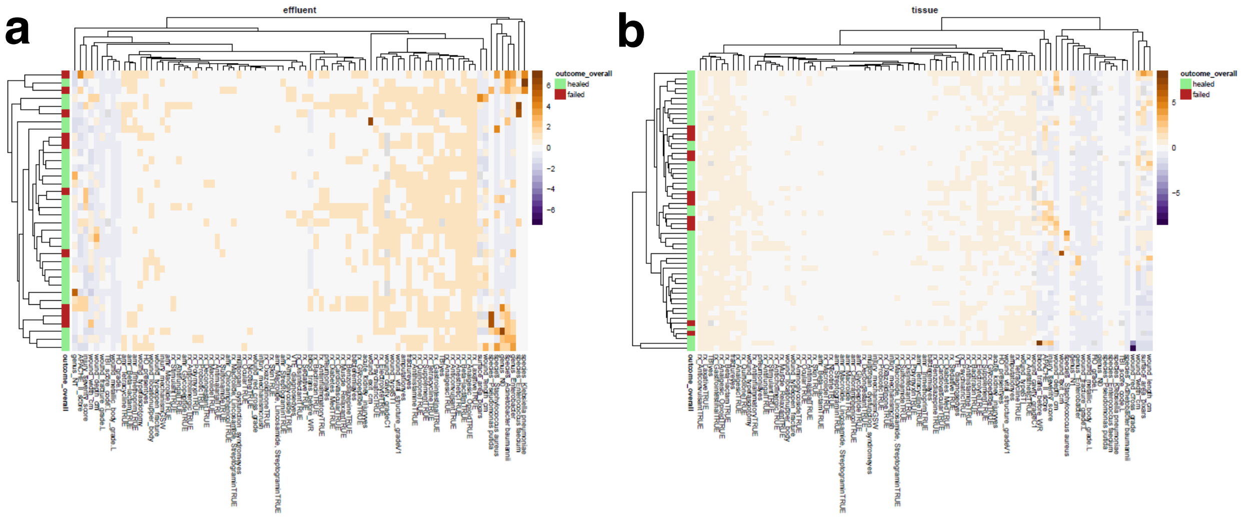


**Supplementary Figure S9. Feature-sample heatmap for variables employed for wound outcome prediction via machine learning classifiers.** Clustering patterns are shown for samples and features that were input to the machine learning classification model training pipeline. Samples are shown in rows and features in columns. Samples are annotated as being derived from healed (green) or failed (red) wounds. Results are shown for samples derived from **A.** effluent and **B.** tissue.

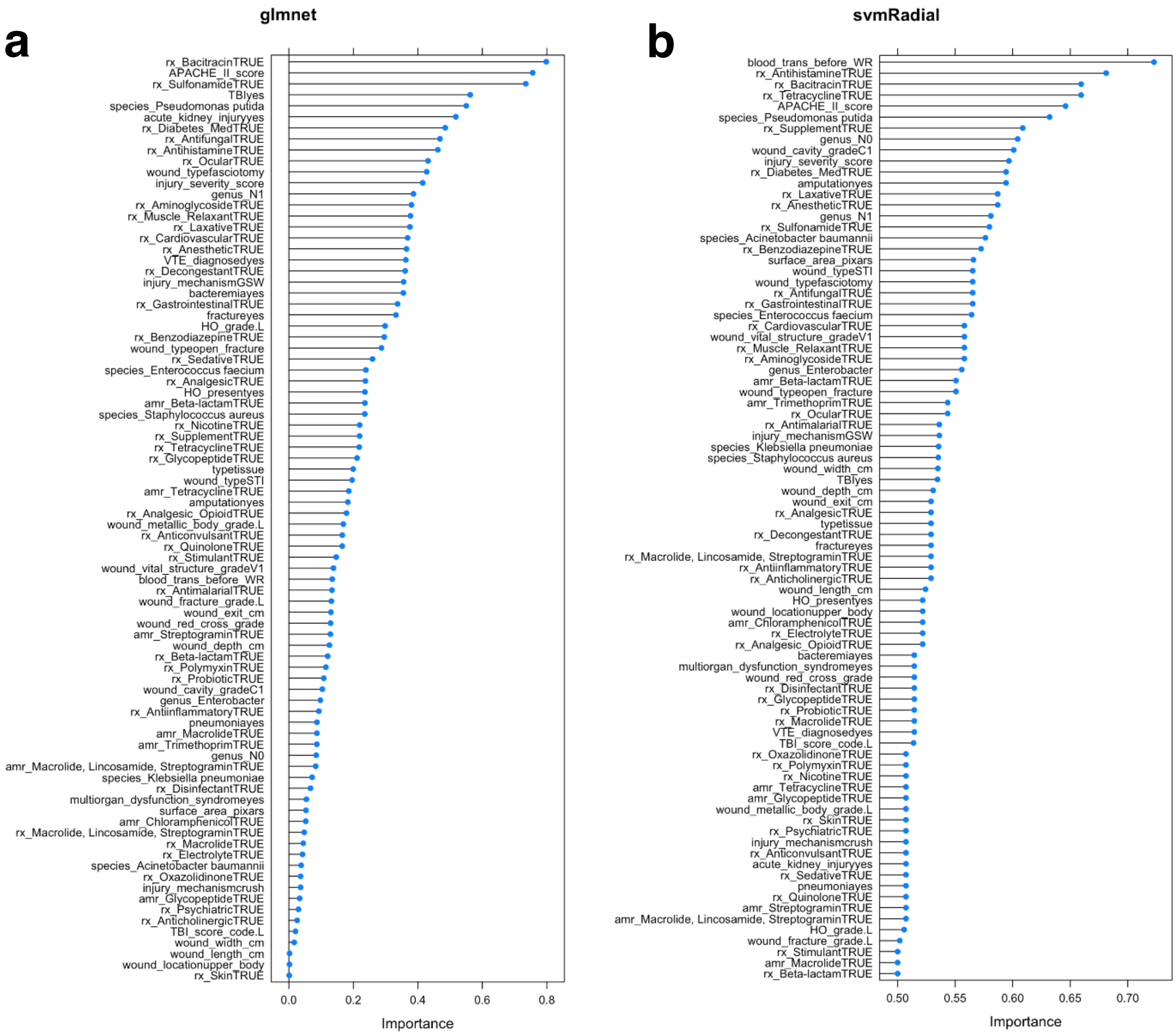

a
glmnet
rx_BacitracinTRUE
APACHE_II_score
rx_SulfonamideTRUE
TBIyes
species_Pseudomonas putida
acute_kidney_injuryyes
rx_Diabetes_MedTRUE
rx_AntifungalTRUE
rx_AntihistamineTRUE
rx_OcularTRUE
wound_typefasciotomy
injury_severity_score
genus_N1
rx_AminoglycosideTRUE
rx_Muscle_RelaxantTRUE
rx_LaxativeTRUE
rx_CardiovascularTRUE
rx_AnestheticTRUE
VTE_diagnosedyes
rx_DecongestantTRUE
injury_mechanismGSW
bacteremiayes
rx_GastrointestinalTRUE
fractureyes
HO_grade.L
rx_BenzodiazepineTRUE
wound_typeopen_fracture
rx_SedativeTRUE
species_Enterococcus faecium
rx_AnalgesicTRUE
HO_presentyes
amr_Beta-lactamTRUE
species_Staphylococcus aureus
rx_NicotineTRUE
rx_SupplementTRUE
rx_TetracyclineTRUE
rx_GlycopeptideTRUE
typetissue
wound_typeSTI
amr_TetracyclineTRUE
amputationyes
rx_Analgesic_OpioidTRUE
wound_metallic_body_grade.L
rx_AnticonvulsantTRUE
rx_QuinoloneTRUE
rx_StimulantTRUE
wound_vital_structure_gradeV1
blood_trans_before_WR
rx_AntimalarialTRUE
wound_fracture_grade.L
wound_exit_cm
wound_red_cross_grade
amr_StreptograminTRUE
wound_depth_cm
rx_Beta-lactamTRUE
rx_PolymyxinTRUE
rx_ProbioticTRUE
wound_cavity_gradeC1
genus_Enterobacter
rx_AntiinflammatoryTRUE
pneumoniayes
amr_MacrolideTRUE
amr_TrimethoprimTRUE
genus_N0
amr_Macrolide, Lincosamide, StreptograminTRUE
species_Klebsiella pneumoniae
rx_DisinfectantTRUE
multiorgan_dysfunction_syndromeyes
surface_area_pixars
amr_ChloramphenicolTRUE
rx_Macrolide, Lincosamide, StreptograminTRUE
rx_MacrolideTRUE
rx_ElectrolyteTRUE
species_Acinetobacter baumannii
rx_OxazolidinoneTRUE
injury_mechanismcrush
amr_GlycopeptideTRUE
rx_PsychiatricTRUE
rx_AnticholinergicTRUE
TBI_score_code.L
wound_width_cm
wound_length_cm
wound_locationupper_body
rx_SkinTRUE
0.0
0.2
0.4
0.6
0.8
Importance
b
svmRadial
blood_trans_before_WR
rx_AntihistamineTRUE
rx_BacitracinTRUE
rx_TetracyclineTRUE
APACHE_II_score
species_Pseudomonas putida
rx_SupplementTRUE
genus_N0
wound_cavity_gradeC1
injury_severity_score
rx_Diabetes_MedTRUE
amputationyes
rx_LaxativeTRUE
rx_AnestheticTRUE
genus_N1
rx_SulfonamideTRUE
species_Acinetobacter baumannii
rx_BenzodiazepineTRUE
surface_area_pixars
wound_typeSTI
wound_typefasciotomy
rx_AntifungalTRUE
rx_GastrointestinalTRUE
species_Enterococcus faecium
rx_CardiovascularTRUE
wound_vital_structure_gradeV1
rx_Muscle_RelaxantTRUE
rx_AminoglycosideTRUE
genus_Enterobacter
amr_Beta-lactamTRUE
wound_typeopen_fracture
amr_TrimethoprimTRUE
rx_OcularTRUE
rx_AntimalarialTRUE
injury_mechanismGSW
species_Klebsiella pneumoniae
species_Staphylococcus aureus
wound_width_cm
TBIyes
wound_depth_cm
wound_exit_cm
rx_AnalgesicTRUE
typetissue
rx_DecongestantTRUE
fractureyes
rx_Macrolide, Lincosamide, StreptograminTRUE
rx_AntiinflammatoryTRUE
rx_AnticholinergicTRUE
wound_length_cm
HO_presentyes
wound_locationupper_body
amr_ChloramphenicolTRUE
rx_ElectrolyteTRUE
rx_Analgesic_OpioidTRUE
bacteremiayes
multiorgan_dysfunction_syndromeyes
wound_red_cross_grade
rx_DisinfectantTRUE
rx_GlycopeptideTRUE
rx_ProbioticTRUE
rx_MacrolideTRUE
VTE_diagnosedyes
TBI_score_code.L
rx_OxazolidinoneTRUE
rx_PolymyxinTRUE
rx_NicotineTRUE
amr_TetracyclineTRUE
amr_GlycopeptideTRUE
wound_metallic_body_grade.L
rx_SkinTRUE
rx_PsychiatricTRUE
injury_mechanismcrush
rx_AnticonvulsantTRUE
acute_kidney_injuryyes
rx_SedativeTRUE
pneumoniayes
rx_QuinoloneTRUE
amr_StreptograminTRUE
amr_Macrolide, Lincosamide, StreptograminTRUE
HO_grade.L
wound_fracture_grade.L
rx_StimulantTRUE
amr_MacrolideTRUE
rx_Beta-lactamTRUE
0.50
0.55
0.60
0.65
0.70
Importance

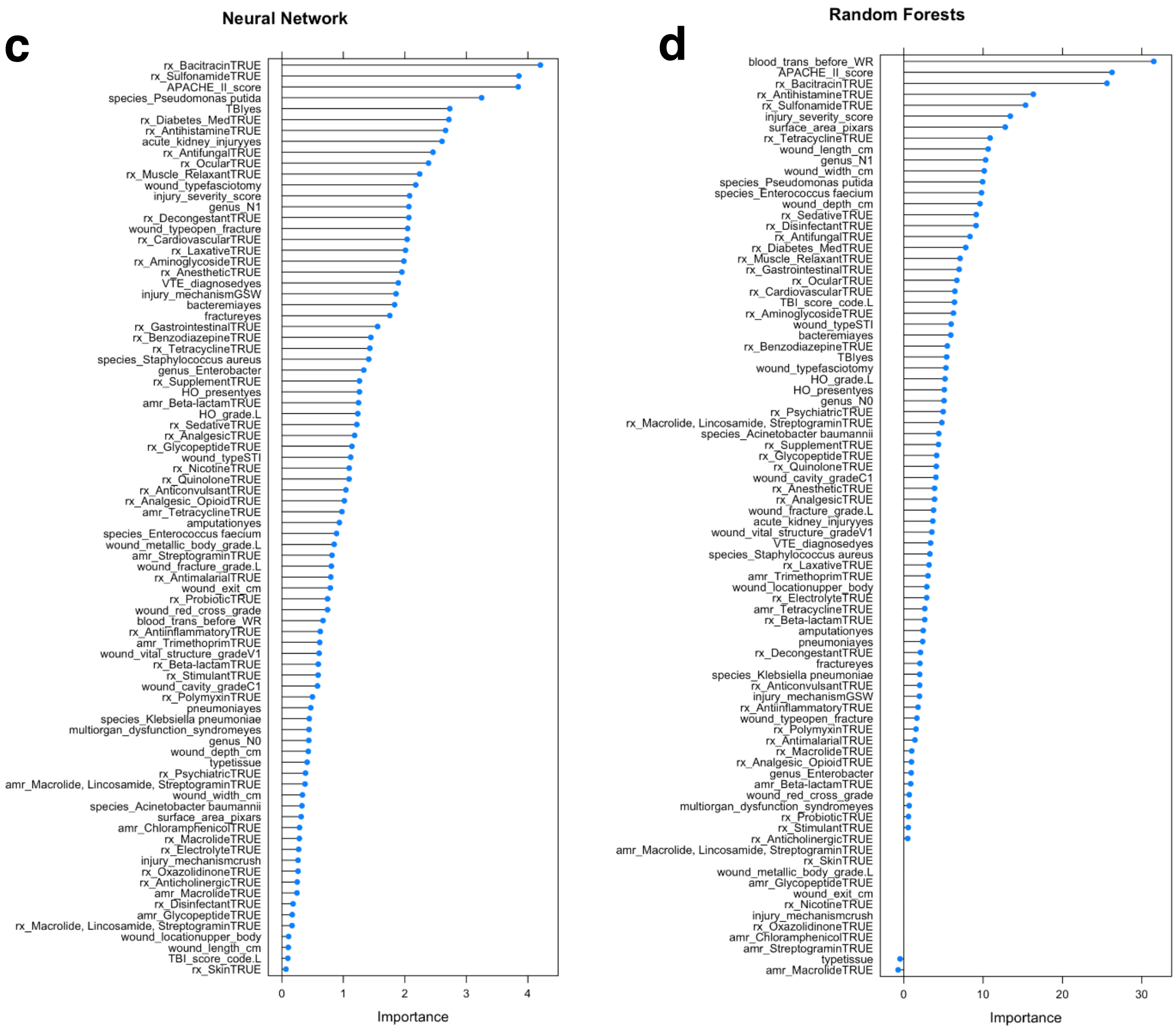


**Supplementary Figure S10. Feature importance plots for machine learning classification models.** Feature importance values are shown for models trained for prediction of wound outcome using all clinical and sequencing-based metagenomic microbial features. For SVM, feature importances given are calculated from the data, independent of the model. The relative importance values for wound and microbial features are shown in rank order. Model classes shown include **A.** penalized logistic regression (glmnet), **B.** model independent (i.e., support vector machine [SVM]), **C.** neural network (NN), and **D.** random forest.

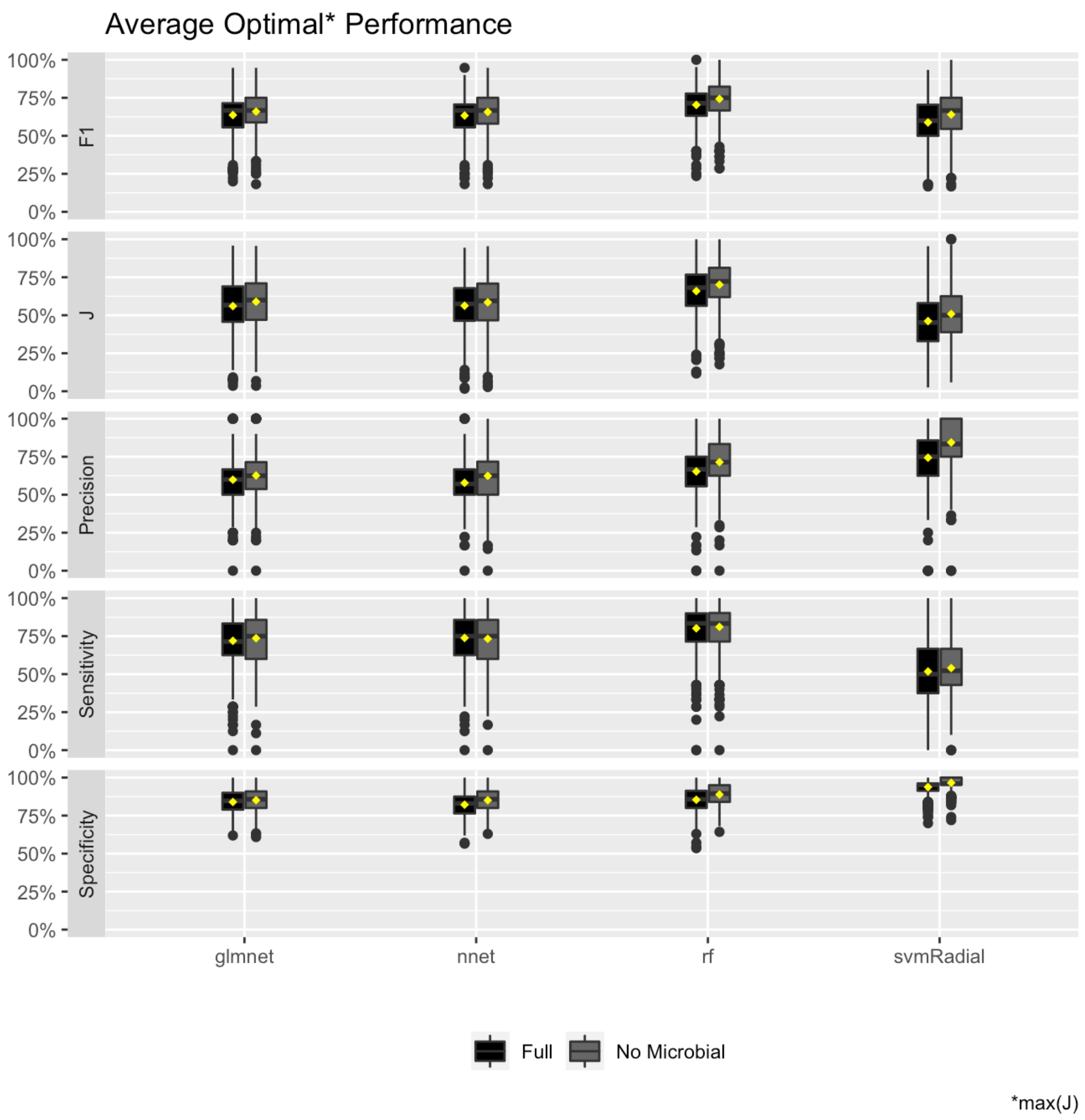


**Supplementary Figure S11. Comparison of performance metrics between feature sets.** Metrics were assessed between machine learning models trained with clinical and metagenomic microbial variables (full) versus clinical variables alone (no microbial).

*Metrics shown for predictions of held-out (aka “out-of-bag”) samples at the threshold that maximizes Youden’s J within each model family’s best tuned model, where model parameters were tuned to maximize area under ROC curve.
